# The genetic structure and demographic history revealed by whole-genome resequencing provide insights into conservation of critically endangered *Artocarpus nanchuanensis*

**DOI:** 10.3389/fpls.2023.1224308

**Published:** 2023-07-27

**Authors:** Changying Xia, Youwei Zuo, Tiantian Xue, Ming Kang, Huan Zhang, Xiaoxia Zhang, Binru Wang, Jiabin Zhang, Hongping Deng

**Affiliations:** ^1^ Center for Biodiversity Conservation and Utilization, School of Life Sciences, Southwest University, Chongqing, China; ^2^ State Key Laboratory of Systematic and Evolutionary Botany, Institute of Botany, Chinese Academy of Sciences, Beijing, China; ^3^ Key Laboratory of Plant Resources Conservation and Sustainable Utilization, South China Botanical Garden, Chinese Academy of Sciences, Guangzhou, China; ^4^ Low Carbon and Ecological Environment Protection Research Center, Chongqing Academy of Science and Technology, Chongqing, China

**Keywords:** *Artocarpus nanchuanensis*, whole-genome resequencing, demographic history, selective sweep, genetic load, conservation unit

## Abstract

**Introduction:**

Whole-genome resequencing technology covers almost all nucleotide variations in the genome, which makes it possible to carry out conservation genomics research on endangered species at the whole-genome level.

**Methods:**

In this study, based on the whole-genome resequencing data of 101 critically endangered *Artocarpus nanchuanensis* individuals, we evaluated the genetic diversity and population structure, inferred the demographic history and genetic load, predicted the potential distributions in the past, present and future, and classified conservation units to propose targeted suggestions for the conservation of this critically endangered species.

**Results:**

Whole-genome resequencing for *A. nanchuanensis* generated approximately 2 Tb of data. Based on abundant mutation sites (25,312,571 single nucleotide polymorphisms sites), we revealed that the average genetic diversity (nucleotide diversity, π) of different populations of *A. nanchuanensis* was relatively low compared with other trees that have been studied. And we also revealed that the NHZ and QJT populations harboured unique genetic backgrounds and were significantly separated from the other five populations. In addition, positive genetic selective signals, significantly enriched in biological processes related to terpene synthesis, were identified in the NHZ population. The analysis of demographic history of *A. nanchuanensis* revealed the existence of three genetic bottleneck events. Moreover, abundant genetic loads (48.56% protein-coding genes) were identified in *Artocarpus nanchuanensis*, especially in genes related to early development and immune function of plants. The predication analysis of suitable habitat areas indicated that the past suitable habitat areas shifted from the north to the south due to global temperature decline. However, in the future, the actual distribution area of *A. nanchuanensis* will still maintain high suitability.

**Discussion:**

Based on total analyses, we divided the populations of *A. nanchuanensis* into four conservation units and proposed a number of practical management suggestions for each conservation unit. Overall, our study provides meaningful guidance for the protection of *A. nanchuanensis* and important insight into conservation genomics research.

## 1 Introduction

The Dalou Mountains, with the famous Jinfo Mountains as the highest peak, are the boundary between the Guizhou Plateau and the Sichuan Basin (López-Pujol et al., 2011; Deng, 2019; Zu et al., 2022; Zu et al., 2023; **Supplementary Figure 1**). Local orogeny led to the formation of its present complex topography, which is mainly composed of low mountain canyons and middle mountain platforms. The humid and warm climate and high habitat heterogeneity together contributed to the abundant biodiversity of the Dalou Mountains. A recent study indicated that there were more than 5,000 species of vascular plants in the Dalou Mountains, including more than 200 plants for which type specimens were collected from the Dalou Mountains (Ding et al., 2014; Deng, 2019). Moreover, due to the Qinling Mountains-Daba Mountains in the north, the Dalou Mountains were less affected by the Quaternary ice age and retained many ancient relict species, such as *Ginkgo biloba*, *Cathaya argyrophylla* and *Taxus chinensis* (Wang and Ge, 2006; Tang et al., 2012; Qian et al., 2016; **Supplementary Figure 1**). Although the Dalou Mountains harbour abundant biodiversity, it has been given less attention than Daba Mountains and Wuling Mountain, which are also located in Chongqing City and designated as priority areas for biodiversity conservation. This has greatly hindered the further protection of endangered plants in this region, especially species with small populations.

Currently, many endangered and narrowly distributed plant species are found in the Dalou Mountains (Deng, 2019). For the rescue of endangered plants, *ex - situ* protection is generally considered to be an important and reliable means to prevent extinction (Huang and Zhang, 2012; Xu and Zang, 2022). However, recent studies have shown that *ex - situ* conservation of endangered plants with a lack of comprehensive population genetic information often leads to failure to cover the genetic diversity of wild populations and achieve protection of germplasm resources (Wu et al., 2013; Xu and Zang, 2022). Therefore, it is very important to identify conservation units for the protection and management of endangered species, which could provide a clear understanding of the distribution of genetic diversity and the boundary of population units for protection managers (Funk et al., 2012; Li et al., 2020; Xu et al., 2022). With recent developments in sequencing technology and reductions in sequencing costs, whole-genome resequencing has become increasingly popular because it covers almost all nucleotide variations in the genome (Hohenlohe et al., 2021; Ma et al., 2021; Ma et al., 2022). This has greatly helped accurately and clearly reveal the demographic history and genetic structure of endangered species, which are further helpful for the division of conservation units and the development of effective conservation strategies (Ma et al., 2021; Ma et al., 2022; Xu et al., 2022).

Climate change is considered to be the major threat to biodiversity in the 21^st^ century because it may lead to loss of biodiversity, termination of evolutionary potential and disruption of ecological services (Thomas et al., 2004; Pimm, 2008; Dawson et al., 2011). For endangered species that are vulnerable to climate fluctuations, it is difficult to formulate appropriate conservation plans without considering the impact of climate change (Huang, 2011). Recent studies have indicated that climate change greatly threatens the population maintenance of endangered species and also drive the distribution pattern of endangered species to change, affecting the effectiveness of species conservation (Ackerly et al., 2010; Barber et al., 2016; Xue et al., 2022). Species distribution modelling combines species distribution records with environmental variables to predict the potential distribution range of species and has been widely used in the assessment of biodiversity conservation and priority management of many endangered species (Qin et al., 2017a; Tang et al., 2018; Gao et al., 2022; Xue et al., 2022).

**Figure 1:**
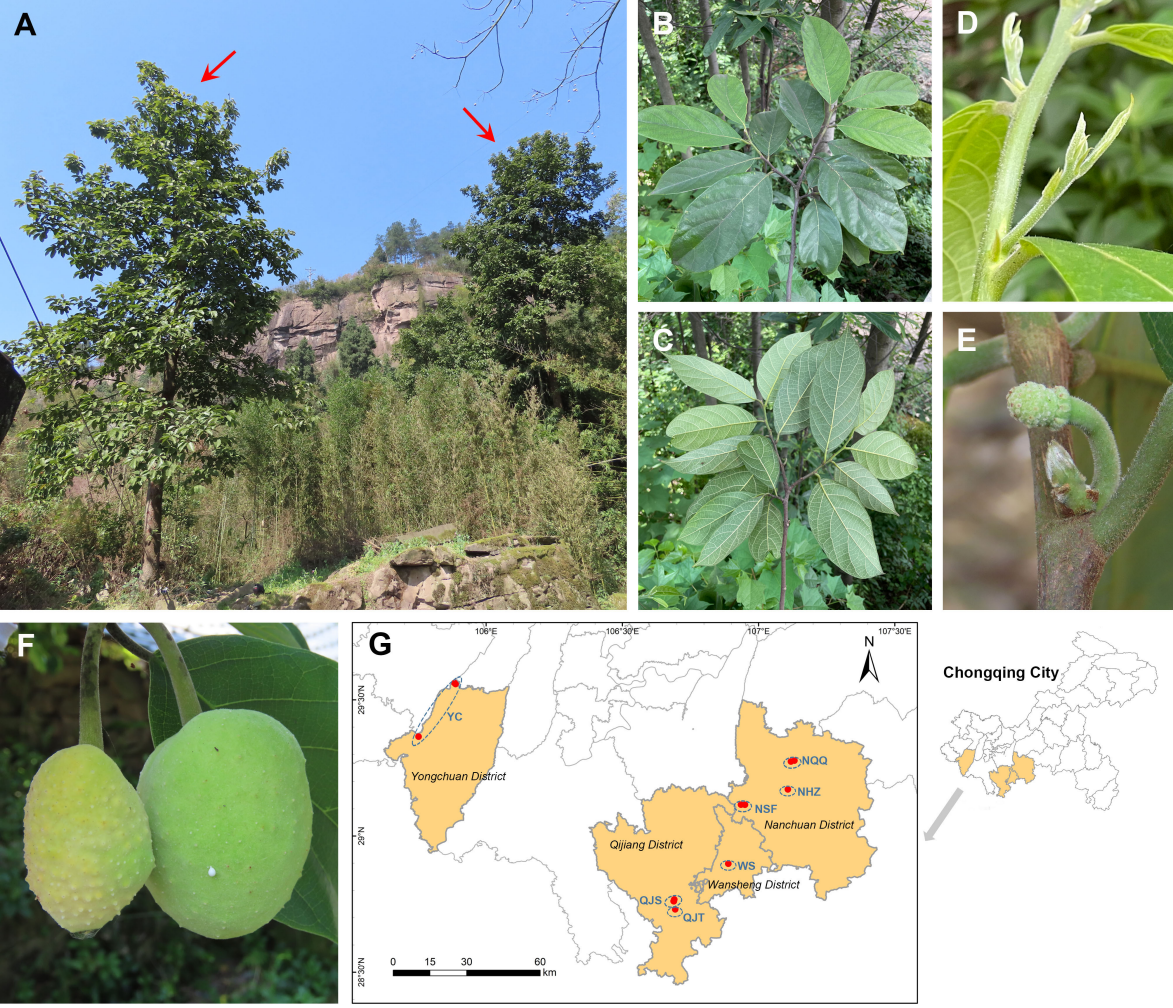
Wild habitats, morphological characters, and geographical distribution of *Artocarpus nanchuanensis*. **(A)** Representative photographs of *A. nanchuanensis*. **(B, C)** Leaves in front view and back view. **(D)** Leaf buds and stipules. **(E)** Inflorescences. **(F)** Fruits. **(G)** Locations of the sampled 7 populations.

*Artocarpus nanchuanensis* S.S. Chang, S.C. Tan, Z.Y. Liu, which is mainly distributed in the northern section of the Dalou Mountains, is the northernmost species in the natural distribution of the genus *Artocarpus* (Wu and Zhang, 1989; **Figure 1**; **Supplementary Figure 1**). This species is distinguished from other species of *Artocarpus* by two rows of alternate leaves, elliptic to nearly round leaves, irregular spherical polythalamic fruit with papillary protrusion on the surface, etc (**Figure 1**). Recent phylogenetic studies have indicated that this species belongs to the basal clade of the subg. *Pseudojaca* (Ser. *Clavati*), which contains mostly Chinese species (Gardner et al., 2021). The fruit of *A. nanchuanensis* is rich in nutrients and can be used for brewing, processing jam and beverages (He, 2014; He et al., 2022a). Straight trunks are often used for shipbuilding and making furniture (He, 2014). Although artificial breeding technologies for *A. nanchuanensis* have gradually been established in recent years owing to its high potential application value, only breeding bases and several parks currently have cultivation (Tan et al., 2015). In addition, due to its fragmented distribution and small wild population, this species was rated as critically endangered (CR) and as a Wild Plants with Extremely Small Populations (WPESP) in Chongqing City (Qin et al., 2017b). A recent field investigation indicated that *A. nanchuanensis* was distributed only in 4 counties of Chongqing City, and no more than 100 maternal trees remained (He, 2014). It was also found that adult trees were rare in most populations, and there was a pattern of more seedlings and fewer young trees (field survey data). Therefore, research on conservation biology and molecular genetics for this critically endangered species is urgently needed to support the formulation of further conservation plans.

The recently reported genome assembly at the chromosome level of *A. nanchuanensis* provided an opportunity to resequence at the whole genome level (He et al., 2022a). To reveal the genetic structure and demographic history of *A. nanchuaensis* fully and accurately and provide meaningful guidance for its conservation, 101 individuals from 7 populations of *A. nanchuanensis* were resequenced in this study. We aimed to: (1) evaluate the genetic diversity and population structure of *A. nanchuanensis*; (2) infer the demographic history of this species and evaluate its genetic load; (3) predict the potential distributions of this species in the past, present and future; and (4) use our analysis results to classify conservation units and propose targeted suggestions for the conservation of this critically endangered species.

## 2 Materials and methods

### 2.1 Sample collection and whole genome resequencing

We collected 101 individuals from 7 populations of *A. nanchuanensis* from Yongchuan District, Qijiang District, Wansheng District and Nanchuan District in southwestern Chongqing City, China (**Figure 1**; **Supplementary Table 1**). Due to the scarcity of adult and young trees of *A. nanchuanensis*, we first sampled all adult and young tree individuals from each population, and then sampled seedlings at intervals of three meters. The number of individuals sampled from each population ranged from 11 to 27. Two populations from Yongchuan District were merged due to following reasons. (1) These two populations are located in the same branch range of Dalou Mountains (**Supplementary Figure 1**). (2) Poorly differentiated and similar genetic backgrounds were detected between them. (3) These two are closest, but far away from populations in other regions.

A tender leaf of each selected individual was collected and taken back to the laboratory for storage at -80 degrees Celsius. Genomic DNA was extracted from samples using the standard cetyltrimethylammonium bromide (CTAB) method (Doyle and Doyle, 1987). A Qubit4 Fluorometer nucleic acid protein fluorescence quantitative instrument and agarose gel electrophoresis were used to detect the concentration and quality of total genomic DNA, respectively. To construct paired-end libraries, we performed following treatments on the total genomic DNA (1 μg) of each sample: fragmentation, end repairing, adaptor ligation, PCR amplification and PCR product cyclization (Huang et al., 2018). The Agilent 2100 Bioanalyzer (Agilent DNA 1000 Reagents) was used to detect the fragment size and concentration of the constructed library. All libraries were sequenced on the BGISEQ-500 sequencing platform, which uses optimized Combinatorial Probe-Anchor Synthesis (cPAS) and DNA nanospheres (DNB) core sequencing technology, producing 150 bp pair end reads (Huang et al., 2018).

### 2.2 Genome mapping and SNP calling

Raw data with adapter sequences or low-quality sequences were filtered using SOAPnuke with the following parameters: -n 0.01 -l 20 -q 0.3 –adaMR 0.25 –ada_trim –polyX 50 –minReadLen 150 (Chen et al., 2018). BWA-MEM (Li, 2013) and default parameters were used to map paired-end clean reads to the reference genome of *A. nanchuanensis* (He et al., 2022b). SAMtools (Danecek et al., 2021) and Picard (http://picard.sourceforge.net/) were used to sort and delete duplicate reads, respectively. Then, we employed the mpileup + call (–multiallelic-caller –variants-only) and filter modules (-g 3 -G 10 -e “%QUAL<10”) in BCFtools (Danecek et al., 2021) to call and filter variable sites in each sample, and the view module (–types snps) in BCFtools was used to extract single nucleotide polymorphisms sites (SNPs) to obtain Dataset 1, including 25,312,571 SNPs. To ensure high-quality SNPs, VCFtools v0.1.17 (Danecek et al., 2011) was utilized to further filter Dataset 1, with the following settings: –maf 0.01 –max-missing 0.9 –min-alleles 2 –max-alleles 2. After filtering, a total of 18,906,649 SNPs (Dataset 2) remained for downstream analysis. SnpEff 5.0e (Cingolani et al., 2012) was used for SNP annotation. We first used SnpEff 5.0e to construct a new database based on the assembly and annotation file of the *A. nanchuanensis* genome, and then annotated Dataset 1 by using the constructed database and default parameters.

### 2.3 Population genetic structure

Principal component analysis (PCA) was conducted by using Plink (Purcell et al., 2007), and the R package (ggplot2; Wickham, 2016) was used for visualization of the analysis results. Population structure was analyzed by Admixture (Alexander et al., 2009), which could infer the stratification relationships of populations. First, PLINK (Purcell et al., 2007) was used to filter the linkage sites and obtain a temporary file containing the sites that needed to be preserved from Dataset 2 with the following parameters: indep pairwise 50 10 0.2. Independent SNPs (Dataset 3) were extracted based on the temporary file and Dataset 2, yielding a total of 5,146,515 SNPs. Then, the bed file converted from the vcf file was used as the input file of Admixture for population structure analysis. According to the total number of natural populations, the number of clusters K was set from 2 to 7. The optimal K value was determined based on the actual distribution information and PCA results. Finally, the results were visualized by Poppelper (Francis, 2017) based on the Q matrix obtained from the Admixture analysis.

### 2.4 Phylogenetic analysis and estimation of population genetic parameters

Based on the latest research of *Artocarpus*, we downloaded the resequencing data of five other species of *Artocarpus* (*A. hypargyreus, A. styracifolius, A. tonkinensis, A. petelottii*, and *A. gomezianus*) to explore the phylogenetic status of *A. nanchuensis* (Gardner et al., 2021; Lin et al., 2022; **Supplementary Table 2**). The first four species are thought to be in the same clade as *A. nanchuensis* (ser. *Clavati*), and the last one belongs to the sisters clade (ser. *Peltati*) of ser. *Clavati* (Gardner et al., 2021). These data were processed with the method described in section 2.2 to obtain SNP data. This dataset, along with Dataset 3 of *A. nanchuanensis*, was used to construct the phylogenetic tree by IQ-TREE (Nguyen et al., 2015). We first converted the vcf file of the combined dataset to phylip format and then used the following settings for tree building: automatic model selection, unpartitioned, and 1000 fast bootstrap analyses. The obtained phylogenetic tree was rooted with *A. gomezianus* (Gardner et al., 2021). In addition, based on the annotation results from SnpEff 5.0e (Cingolani et al., 2012), we calculated the genetic diversity of synonymous (π_S_) and nonsynonymous sites (π_N_) of each population using VCFtools with a 100-kb sliding window and a step size of 10 kb. Genetic diversity was defined as the average number of pairwise nucleotide differences (nucleotide diversity, π) in this study (Nei and Li, 1979; Tajima, 1983; Corbett-Detig et al., 2015). VCFtools v0.1.17 (Danecek et al., 2011) was also used to calculate other population genetic parameters with a 100-kb sliding window and a step size of 10 kb based on Dataset 2, including fixation statistics (*F_ST_*) and π ratio values between each population pair.

### 2.5 Selective sweep analysis

To identify the potential selective sweeps in the two special populations of *A. nanchuanensis* (NHZ and QJT), we selected the windows with high genetic differentiation (top 1% of *F_ST_* and top 1% of nucleotide diversity ratio values (π_QJT_/π_NHZ_)) as the candidate divergent regions (CDRs) by using Microsoft Excel. Candidate genes were obtained from CDRs and GO enrichment analysis was carried out by using TBtools (Chen et al., 2020). Then we extracted the main genes from the significantly enriched GO items and analyzed the clustering of these genes in the corresponding gene family. The complete members of the gene family in *A. nanchuanensis* were searched and confirmed by HMMER 3.0 (Finn et al., 2015) and the NCBI Conserved Domain database (https://www.ncbi.nlm.nih.gov/Structure/cdd/wrpsb.cgi). In addition, the genes of the corresponding gene family in *Arabidopsis thaliana* were downloaded from the Phytozome database (Goodstein et al., 2012) and used together with the genes of *A. nanchuanensis* to construct a phylogenetic tree.

### 2.6 Demographic history inference

To reconstruct the detailed demographic history of *A. nanchuanensis*, BCFtools (mpileup + call module) (Danecek et al., 2021) was used to obtain the consensus sequence between the sample and reference genome, and the fq2psmcfa module embedded in paired sequential Markov condensation (PSMC) (Li and Durbin, 2011) was used to convert its output file into fasta-like format. Then, the demographic history of each population was inferred by a PSMC model (Li and Durbin, 2011) with the following parameter settings: - N25 - t15 - r5 - p “4 + 25 * 2 + 4 + 6. The generation time was set to 11 years on the basis of years of field observation of *A. nanchuanensis*, and the mutation rate per generation was set to 2.86e-8 (11* 2.6e-9) (Gaut et al., 1996).

### 2.7 Characteristics of deleterious mutations

It is predicted that deleterious mutations (genetic load) will destroy gene function, which would significantly reduce the fitness of individuals in the population (Mattila et al., 2012). Moreover, loss of function (LOF) was the major type of deleterious mutations. To estimate the genetic load of *A. nanchuanensis*, we first defined the dominant alleles in all individuals (more than 50% of individuals had homozygous alleles) as the ancestral allele genotype (Hu et al., 2021). Then, the ancestral allele loci of each individual were extracted and annotated by using VCFtools v0.1.17 (Danecek et al., 2011) and SnpEff 5.0e (Cingolani et al., 2012), respectively. For each individual, we calculated the number of homozygous LOF sites and the number of genes containing one or more homozygous LOF sites divided by the total number of protein-coding genes (PCGs). Finally, gene ontology (GO) analysis was applied to characterize the detected genes containing homozygous LOF sites based on the genome annotation results of recent studies by using TBtools (Chen et al., 2020; He et al., 2022a).

### 2.8 Ecological niche modelling

To further reveal the change in the distribution pattern of *A. nanchuanensis*, MaxEnt (version 3.3) (Phillips et al., 2006) was used to predict the past (mid Pliocene warm period - ca. 3.205 Ma, and MIS19-ca. 787 ka), current (1979-2013) and future (2070 years, RCP4.5) potential suitable habitat areas. Since five bioclimatic variables were missing in the two past periods, only 14 bioclimatic variables were downloaded for the prediction (**Supplementary Table 3**). The 14 bioclimatic variables of current climate data and paleoclimate data were downloaded from the Paleoclim database (Brown et al., 2018) with 2.5 min resolution. The future climate data (2070, BCC-CSM1-1 model, RCP 45) were downloaded from the WorldClim database (https://worldclim.org/data/v1.4/cmip5_2.5m.html). To minimize the impact of multicollinearity and overfitting on the stability and quality of the model, the 14 bioclimatic variables were analyzed with the Pearson correlation test. When the correlation coefficient was > |0.80|, one of the two variables was removed. Ultimately, six bioclimatic variables were selected to predict the suitable habitat areas for four time periods, including BIO1 (annual mean temperature), BIO4 (temperature seasonality), BIO8 (mean temperature of wettest quarter), BIO12 (annual precipitation), BIO14 (precipitation of driest month), and BIO15 (precipitation seasonality; **Supplementary Table 3**). We obtained 23 distribution records from the field investigations, which covered most of the known living areas of *A. nanchuanensis* (**Supplementary Table 4**). We also removed duplicate records in the same grid cell at a spatial resolution of 2.5 arc minutes to reduce spatial autocorrelation (Boria et al., 2014). Finally, a total of 18 distribution records were used in the analysis. In addition, we extracted the six bioclimate variables from these 18 records for principal component analysis (R package). The first two principal components (PC1 and PC2) were extracted from the analysis to estimate the main changes in bioclimatic variables represented by climate instability.

## 3 Results

### 3.1 Whole-genome resequencing

The whole-genome resequencing of 101 individuals generated approximately 2 Tb of data. The average coverage, average depth and primary mapping rate of all samples were 98.00%, 39.23 and 97.57%, respectively (**Supplementary Table 5**). The annotation results of all SNPs indicated that 60.80% of the SNPs were located in intergenic regions, 13.12% were located in exon regions, and 8.18% were located in intron regions (**Supplementary Table 6**).

### 3.2 Population structure

**Figure 2:**
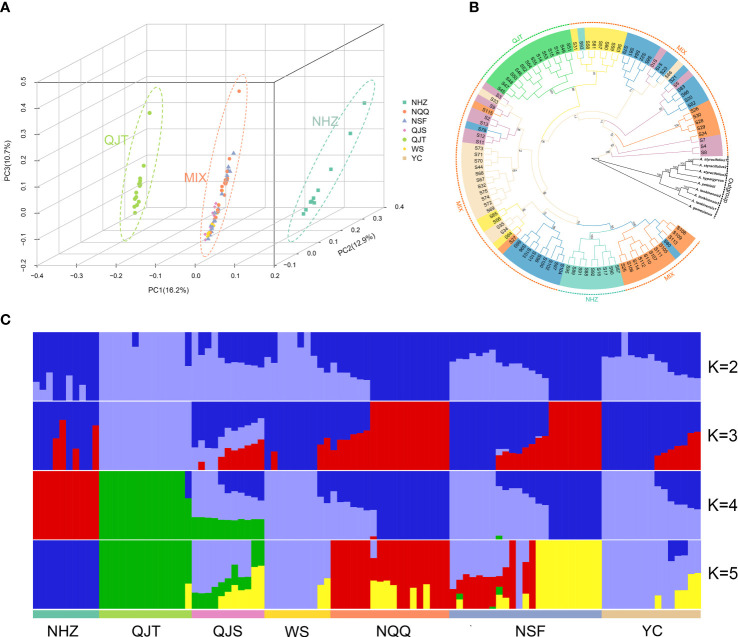
Genetic structure and phylogeny of *Artocarpus nanchuanensis*. **(A)** Principal component analysis (PCA) plot for the 101 *A. nanchuanensis* individuals based on PC1, PC2, and PC3. **(B)** Phylogenetic tree based on 101 *A. nanchuanensis* individuals and outgroups, with numbers on branches representing bootstrap percentages. Different colours of the branches represent individuals of *A nanchuanensis* from different populations. **(C)** Population structure of 101 *A. nanchuanensis* individuals based on K=2-5.

The PCA indicated that the first three principal components (PCs) explained 16.2%, 12.9% and 10.7% of the total genetic variance, respectively. PC1, PC2, and PC3 significantly separated the NHZ and QJT populations from all other populations, but did not separate the remaining five populations (**Figure 2A**; **Supplementary Figures 2**, **3**). The analysis of population structure showed that for the optimal K value (K=4), all samples were divided into four groups, including QJT, NHZ, QJS and four other populations (**Figure 2C**). It is worth noting that the genetic backgrounds of the NHZ and QJT populations were quite simple, with only a few genetic mixings compared to other populations (**Figure 2C**). The QJS population, which was geographically close to the QJT population (only separated by a 30-meter-wide river), had a distinct genetic mixture of QJT and the other four populations (NQQ, NSF, WS and YC). However, there was no significant difference in genetic background among the four populations from Nanchuan District, Wansheng and Yongchuan District (NQQ, NSF, WS and YC) (**Figure 2C**). The *F_ST_* analysis indicated that the degrees of differentiation between various populations of *A. nanchuanensis* were relatively low, all less than 0.01 (**Supplementary Table 7**).

### 3.3 Phylogenetic and genetic parameter estimation

Phylogenetic analysis showed that *A. nanchuanensis* and four species of ser. *Clavati* (*A. tonkinensis*, *A. petelotii, A. hypargyreus*, and *A. styracifolius*) gathered together and formed a sister branch (**Figure 2B**). Consistent with the PCA results, the NHZ and QJT populations were significantly separated from the other populations in the phylogenetic tree, while the remaining five populations (MIX) were mixed to varying degrees (**Figure 2B**). In addition, the average genetic diversity of different populations of *A. nanchuanensis* was relatively low, with π_S_ values ranging from 1.3×10^-3^ to 1.33×10^-3^ and π_N_ values ranging from 2.26×10^-3^ to 2.32×10^-3^, and there were no significant differences between populations (**Supplementary Table 8**).

### 3.4 Signals of selective sweeps between populations

**Figure 3:**
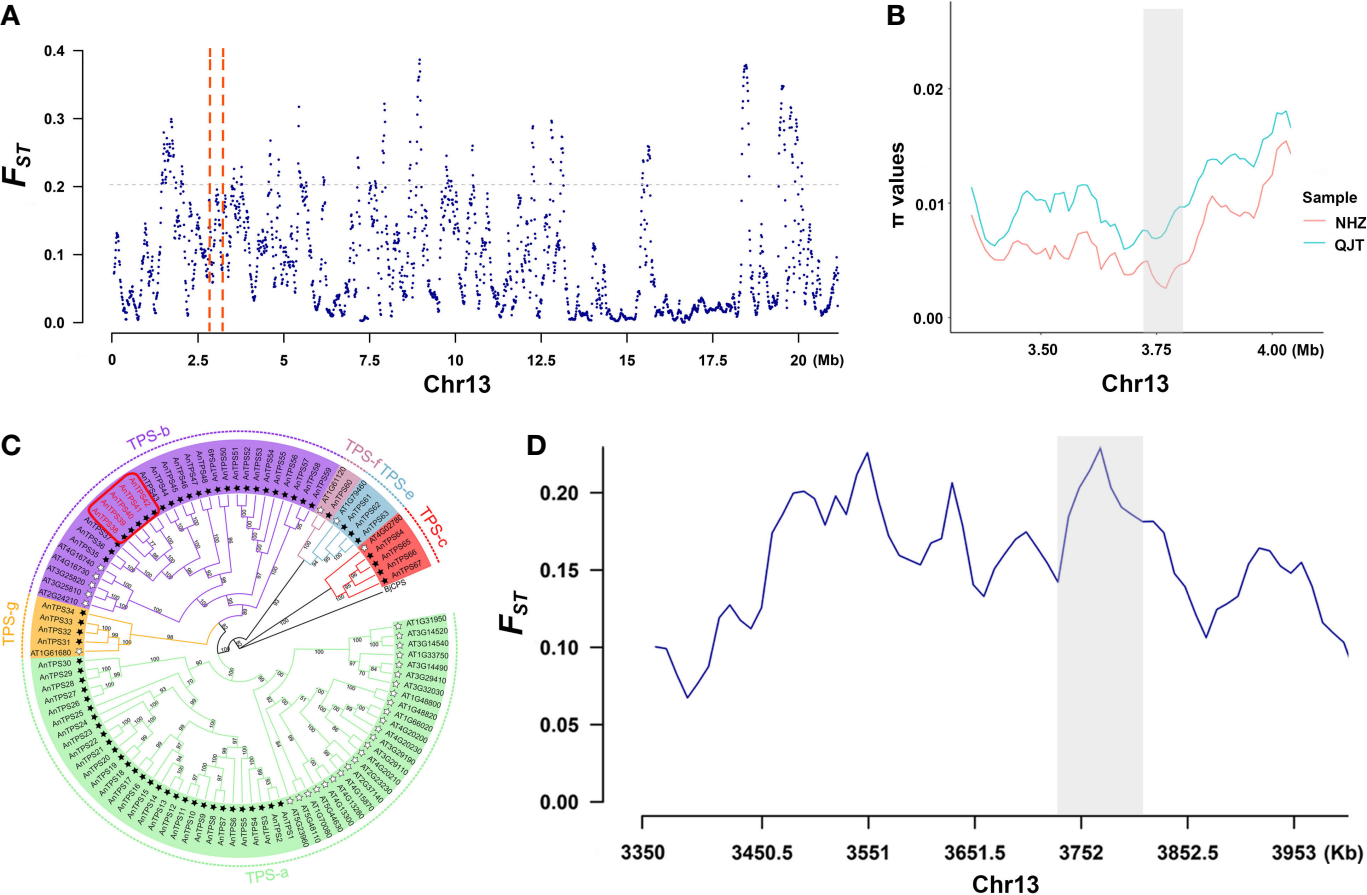
Signals of selective sweep between QJT and NHZ. **(A)** Genomic landscape of selection signals on chromosome 13 between QJT and NHZ. Vertical red dashed lines represent the 500-kb region around these 5 *TPS* genes. **(B)** π values of 5 *TPS* genes linking region between QJT and NHZ. Red line indicates the nucleotide diversity of NHZ population. Green line indicates the nucleotide diversity of QJT population. The gray shadow represents the location of 5 *TPS* genes. **(C)** Phylogenetic analysis of *TPS* gene families in *Artocarpus nanchuanensis* and *Arabidopsis thaliana*. The names of subfamilies are shown outside of the circle. Black star refers to *AnTPSs* and white star refers to the *AtTPSs*. The red rectangular box indicates the locations of five significantly enriched TPS genes. **(D)** *F_ST_* of 5 *TPS* genes linking region between QJT and NHZ. The gray shadow represents the location of 5 *TPS* genes.

In the selective sweeps analysis, we detected 376 genes of 375 CDRs that were under clear positive selection in the NHZ population (**Supplementary Table 9**; **Supplementary Table 10**). These genes were significantly enriched in biological processes related to the synthesis of terpenes (**Supplementary Figure 5**), mainly involving five terpene synthase (*TPS*) genes (*TPS38, TPS39, TPS40, TPS41, and TPS42*) (**Figure 3**; **Supplementary Table 11**). The phylogenetic tree constructed based on 57 detected *TPS* genes of *A. nanchuanensis* and 33 *TPS* genes of *Arabidopsis thaliana* indicated that these five *TPS* genes belong to the *TPS*-b subfamily, and they were notably clustered into a small clade with high bootstrap support (bootstrap percentage 100%; **Figure 3C**).

### 3.5 Demographic history of *A. nanchuanensis*

**Figure 4:**
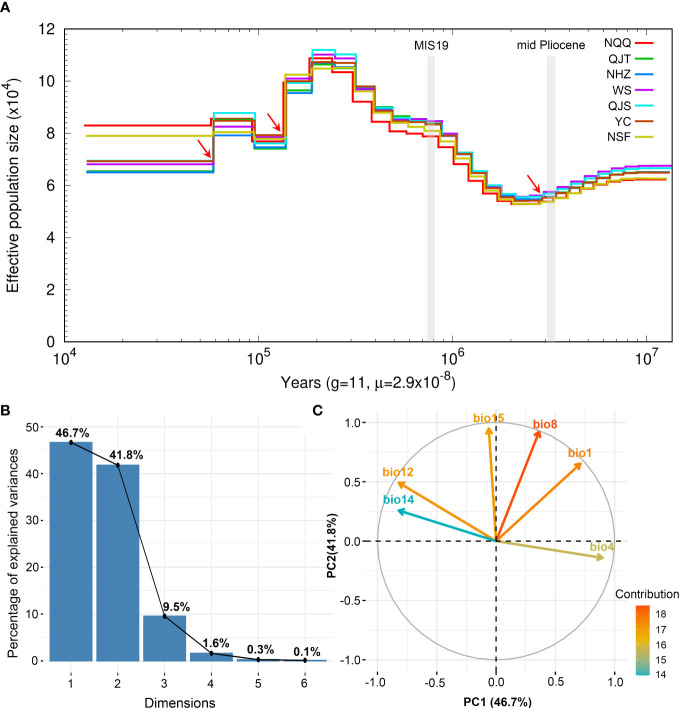
Demographic history of *Artocarpus nanchuanensis* and PCA results of six bioclimatic variables representing climate lability of *A. nanchuanensis*. **(A)** The historical changes in effective population size (Ne) for *A. nanchuanensis* revealed by PSMC analysis, with a generation time (g) of 11 years, and a mutation rate (μ) of 2.9e-8 per site per generation. The red arrows indicate genetic bottleneck events. **(B)** Scree plot of the percentage of explained variance. **(C)** PCA biplot of contribution of six bioclimatic variables to first two PCs.

The results of PSMC analysis indicated that *A. nanchuanensis* experienced three genetic bottleneck events in its evolutionary history (**Figure 4A**). The effective population size (N_e_) of *A. nanchuanensis* began to decline slowly from 10 Ma and encountered the first genetic bottleneck at 2.82 Ma. From 2.2-0.2 Ma, the N_e_ gradually expanded from 5.4x10^4^ to 1.12x10^5^. However, the N_e_ has been declining since 0.19 Ma, except for a small increase at 0.09 Ma. Except for the earlier first genetic bottleneck, this species experienced the second and third genetic bottlenecks at 0.14 Ma and 0.06 Ma, respectively. Finally, the N_e_ of *A. nanchuanensis* was stable in the range of approximately 6.5 x10^4^ - 8.3x10^4^.

### 3.6 Estimation of deleterious mutations

Loss of function in a homozygous state could be used as an indicator of species fitness. The statistical analysis of the annotation results for each individual revealed no significant differences in genes containing homozygous LOF sites/PCGs between populations (**Supplementary Table 12**). The mean value for all individuals was 48.56%. A total of 285 GO terms were assigned in the enrichment analysis of genes containing homozygous LOF sites, including 220, 34 and 31 GO terms belonging to the biological process, molecular function and cell component categories, respectively (**Supplementary Table 13**). In the biological process category, multiple GO terms related to plant development in early stages, as well as potassium ion homeostasis (GO: 0055075) and tyrosine catalytic process (GO: 006572), were significantly enriched (corrected p value < 0.05, **Supplementary Figure 6**).

### 3.7 Niche modelling

**Figure 5:**
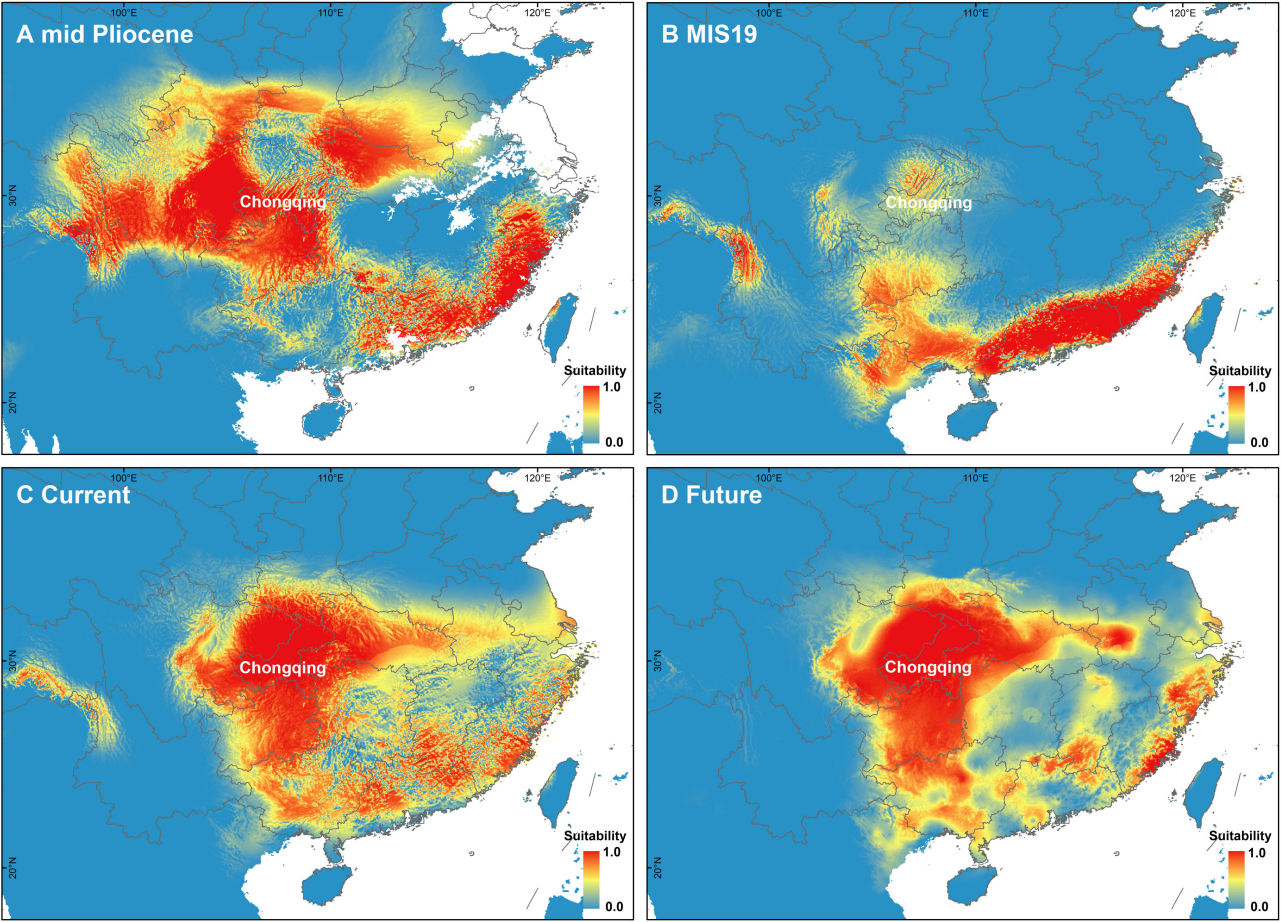
Distribution patterns of potential suitable habitat areas. **(A)** Potential suitable habitat areas under the paleoclimate scenario of mid-Pliocene warm period (3.205 Ma). **(B)** Potential suitable habitat areas under the paleoclimate scenario of MIS19 (ca. 787 ka). **(C)** Potential suitable habitat areas under the current climate scenario (1979-2013). **(D)** Potential suitable habitat areas under the future climate scenario (2070).

The PCA of bioclimate variables showed that the first two PCs (PC1 and PC2) explained 88.5% of the total climate change variation experienced by *A. nanchuanensis* (**Figure 4B**; **Supplementary Figures 7**, **8**). Among the six bioclimatic variables, mean temperature of the wettest quarter (BIO8) and annual average temperature (BIO1) contributed most significantly to the first two PCs, followed by annual precipitation (BIO12) and precipitation seasonality (BIO15) (**Figure 4B**; **Supplementary Figure 9**). The prediction results showed that the area under the operating characteristic curve (AUC) of the model used in the analysis was 0.998, indicating that the prediction model had reached an excellent threshold (Swets, 1988). This suggested that the model employed in this study could accurately describe the relationship between species and climate. The results of the prediction analysis showed that in the mid-Pliocene warm period (3.205 Ma), the suitable habitat areas of *A. nanchuanensis* were mainly distributed in central and south-eastern China (**Figure 5A**). However, the suitable habitat areas were predicted to shift from northern to southern China and were mainly distributed in the south parts of China in MIS19 (Pleistocene, ca. 787 ka; **Figure 5B**). The prediction results also indicated that the current suitable habitat areas, which were widely distributed in central and south-eastern China, were more extensive than the actual distribution (**Figure 5C**). Although the future suitable habitat areas were predicted to shrink slightly compared with the current suitable habitat areas, the actual distribution area of *A. nanchuanensis* (Chongqing City) will still maintain high suitability (**Figures 5C, D**).

## 4 Discussion

In this study, we sampled 101 individuals of *A. nanchuanensis*, covering most of the wild distribution areas, and revealed its complex population structure, demographic history, and genetic threats it currently faces by using whole-genome resequencing data. In addition, we identified several key factors that led to the formation of the distribution pattern of *A. nanchuanensis* by combining the analysis of niche models and demographic history. Our findings provide important guidance for meaningful conservation actions for this critically endangered WPESP.

Compared to that of other trees that have been studied, the genetic diversity of *A. nanchuanensis* was lower (Corbett-Detig et al., 2015; Hazzouri et al., 2015; Wang et al., 2016; Lin et al., 2022), which may be due to the following factors: (1) Previous studies have shown that genetic bottlenecks may lead to a large loss of genetic diversity due to enhanced genetic drift and a reduction in natural selection efficiency (Jangjoo et al., 2016; Ma et al., 2021; Ma et al., 2022). This seemed to be reasonable in the case of *A. nanchuanensis* because the demographic history showed three potential bottleneck events, which may have led to a significant reduction in its genetic diversity. (2) The actual N_e_ found in the field investigation (<200) was significantly smaller than the N_e_ (>65,000) inferred from demographic history, indicating that the population of *A. nanchuanensis* has recently encountered strong pressure. During our field investigation in 2019, three small populations of *A. nanchuanensis* were found in Qianqiu village (Nanchuan district). When we visited the site again in 2022, we found that only a few young trees remained in one of the populations. The main reason was that its straight trunks of *A. nanchuanensis* are often used by local people for shipbuilding and furniture. Based on the inferred large N_e_ value and the evidence that multiple existing populations in the four districts shared very similar genetic backgrounds, we speculated that *A. nanchuanensis* could be widely distributed in the mountains around the four districts. However, this may not be the case, possibly because human activities such as deforestation, road construction, and the expansion of farmland have drastically reduced the N_e_ of *A. nanchuanensis*. Human interference and habitat fragmentation will further accelerate the impact of genetic drift, thereby reducing genetic diversity (Ma et al., 2021). (3) Studies on other *Artocarpus* species indicated that birds and large mammals may be potential seed disseminators (Dickinson et al., 2020). However, considering that most of the existing populations of *A. nanchuanensis* are located near settlement, the likelihood of the occurrence of large mammals is extremely low. Moreover, field investigations showed that mature individuals of *A. nanchuanensis* are rare (numbers between 1 and 5) in most populations, and the geographical distances between populations usually exceed 10 kilometers. These factors may together hinder the production of outbred seeds, which may be an important way to increase the genetic diversity of the population.

*Artocarpus* is a genus mainly distributed in tropical regions, and its diversity center is located in Borneo (Gardner et al., 2021). However, as the northernmost species of this genus, the reason for the formation of the distribution pattern of *A. nanchuanensis* has always been a mystery. Our study revealed that Quaternary climate change played an important role in forming the current distribution pattern of this species, especially temperature. A recent study indicated that the global temperature has been falling since 10 Ma (Westerhold et al., 2020). Combining the prediction analysis and demographic history, we speculated that due to the decrease of temperature in the early stage (10-2.82 Ma), the suitable habitat areas of *A. nanchuanensis* shrank, accompanied by a slow decline in N_e_ (**Figures 4A**, **5A, B**). With the advent of the Quaternary Ice Age, most areas of China were covered by glaciers, except for the mountainous areas in central and southern China, which harboured diverse terrain and a suitable climate, providing long-term stable habitats for plants (Axelrod et al., 1998; López-Pujol et al., 2011; Tang et al., 2012). Thus, *A. nanchuanensis* might have undergone migration and expansion in this region (south of the Qinling-Daba Mountains) during most of the Pleistocene (2.2-0.2 Ma) (**Figures 4A**, **5B**). However, N_e_ began to decline rapidly at 0.19 Ma, which coincided with the penultimate glaciation (0.19-0.14 Ma) (Colleoni et al., 2016). In general, during the Pleistocene glaciation (2000-12ka), the significant increase and decrease of Ne detected in *A. nanchuanensis* was consistent with the published research on other Spermatophytes and *Ginkgo biloba*, which is located in the same refuge - Dalou Mountains (Qiu et al., 2011; Zhao et al., 2019; Ma et al., 2021; Ma et al., 2022). In addition, the recent comparative analysis of demographic history of *Artocarpus* species showed that bioclimatic change and habitat were important factors in shaping the population history (Patil et al., 2022). Therefore, we inferred that similar habitat - acid soil of *A. nanchuanensis* and *A. altilis* may partly explain their highly consistent demographic histories (Patil et al., 2022).

The prediction results showed that the current suitable habitat areas of *A. nanchuanensis* were far larger than its actual distribution area (**Figure 5C**), which may be due to the rapid climate fluctuations in the late Quaternary (approximately 0.8 - 0.1 Ma, Hofreiter and Stewart, 2009), leading to the disappearance of populations in other areas, while only the populations in the refuge could survive. Previous studies have shown that the Dalou Mountains are an important refuge for plants, harbouring many living fossil plants, such as *Ginkgo biloba*, *Cathaya argyrophylla*, and *Davidia involucrata*. (Wang and Ge, 2006; Tang et al., 2012). Therefore, we speculated that only the remaining population of *A. nanchuanensis* located in Dalou Mountain survived in the face of the considerable challenges of climate change. In addition, little is known about the pollination and diffusion mechanisms of *A. nanchuanensis*, and further research is urgently needed in the future to comprehensively infer the underlying reasons for the differences between the current suitable habitat areas and actual distribution area. It is also worth noting that our prediction analysis did not include environmental variables, such as topographical variables and elevation, which should be integrated to yield more accurate prediction results in the future.

The GO annotation of positive selection signal regions of *A. nanchuanensis* indicated that the NHZ population was significantly enriched in 5 TPS genes, which were detected to have a close genetic relationship (**Figure 3**). TPS is the key enzyme responsible for terpene biosynthesis (Yu et al., 2020; Jia et al., 2022), which plays an important role in the defense mechanism of plants against herbivores and pathogenic microorganisms (Kessler, 2001; Gershenzon and Dudareva, 2007; Richter et al., 2015) and in the process of attracting pollinators (Yu et al., 2020; Zhao et al., 2021). There are only two adult trees and dozens of annual seedlings in the NHZ population, one of which is a listed ancient tree (DBH 63 cm), and the other adult tree (DBH 25 cm) is inferred as its offspring. This population is relatively isolated and now surrounded by cities (located within the crematorium area in Nanchuan District). Combined with the unique genetic background of the NHZ population revealed by the population structure analysis, it is inferred that the population has undergone a certain degree of differentiation during the long-term isolated growth process, especially in defense-related genes.

Our study also showed that there is a large amount of genetic load in *A. nanchuanensis* (Hu et al., 2020; Yi et al., 2022; **Supplementary Table 12**). Moreover, the annotation of genes containing homozygous LOF sites of all populations revealed that they were significantly enriched in multiple GO terms related to plant development in the early stage and plant immunity, such as potassium ion homeostasis (GO: 0055075) and tyrosine catabolic process (GO: 006572; **Supplementary Figure S6**). Previous studies have shown that potassium (K+) plays an important role in many basic processes in plants, including cell homeostasis, membrane transport, osmotic regulation and the immune response (Amtmann et al., 2008; Wang and Wu, 2015; Shi et al., 2018). Tyrosine phosphorylation has also been shown to be crucial for regulating immune signals in plants (Lin et al., 2014; Macho et al., 2014; Macho et al., 2015; Liu et al., 2018). All of these GO terms were related to deleterious mutations, which may reduce the ability of *A. nanchuanensis* to adapt to unsuitable environments. Field observations indicated that most populations included numerous newly sprouted seedlings, but perennial seedlings or saplings were rare. Therefore, it could be inferred that the seed germination rate of *A. nanchuanensis* is high, but the seedling survival rate is low, which may be related to the accumulation of deleterious mutations in its genes about development or immunity. However, further analysis of these deleterious mutations is needed to reach a definite conclusion.

Most of the populations of *A. nanchuanensis* are located outside nature reserves. In addition, the low genetic diversity and high genetic load, as well as serious human destruction and habitat disturbance, mean that the future survival of *A. nanchuanensis* may face great challenges. Based on our study results and actual conservation status, we propose to divide all populations into four conservation units, NHZ, QJT, QJS and four other populations (WS, NSF, NQQ and YC), and implement the following protection actions as soon as possible: (1) PCA, genetic structure and phylogenetic analysis revealed the unique genetic backgrounds of QJT and NHZ, which were different from those of other populations (**Figures 2A, C**). To protect the genetic diversity of *A. nanchuanensis*, priority should be given to the protection of these two small populations. The geographical distance between QJT and QJS was only 1 km, with the populations separated by a river approximately 30 meters wide. Surprisingly, our analysis showed that QJT and QJS had significant similarities and differences in genetic composition at the same time, which seemed to be an excellent example of geographical isolation and differentiation (**Figures 2A, C**). Therefore, we suggest establishing plant microreserves for these two small populations in Qijiang District, which harbour many apparent advantages, including lower costs, fast construction, and strong pertinence, and could also improve the public awareness of biodiversity conservation of *A. nanchuanensis* among local residents and even society (Fos et al., 2014; Laguna et al., 2016; Zhang, 2017). The NHZ population is restricted to flower beds of the crematorium in Nanchuan District, and one of the adult trees is listed as an ancient tree. Therefore, consulting with the management personnel of the crematorium is recommended to increase fencing for the flower beds, allowing this population to grow and breed naturally without human interference.

(2) Our field investigation revealed that the individual with the largest DBH (65 cm vs. 63 cm of the listed ancient tree from NHZ) was located in Honglu Town, Yongchuan District, and there is a sand factory under construction around the ancient individual. It is recommended to contact the local forestry administration to confirm the age of the ancient tree as soon as possible and apply *in situ* conservation for this small population with only three mature trees. (3) Considering the rarity of adult trees, isolated populations and small *F_ST_* values between various populations of *A. nanchuanensis*, artificial cross-pollination among four populations with similar genetic backgrounds should be carried out to maintain the genetic diversity and increase the survival potential of this species. (4) Based on the current status of the extremely small population of *A. nanchuanensis*, it is strongly recommended to pursue ex situ protection of the populations of the four conservation units to comprehensively preserve the germplasm resources and genetic diversity of this species. Considering the current and future habitat suitability of *A. nanchuanensis*, large-scale botanical gardens in central China are the preferred choices, such as the Nanshan Botanical Garden in Chongqing City and the Wuhan Botanical Garden of the Chinese Academy of Sciences in Hubei Province.

## 5 Conclusion

Based on the whole-genome resequencing data of 101 critically endangered *A. nanchuanensis* individuals, we revealed that repeated bottleneck events, human disturbance and habitat fragmentation, as well as excessive self-pollination within populations, together contributed to low genetic diversity. Quaternary climate change, especially temperature change, plays an important role in forming the current distribution pattern and endangered status of the species. The Dalou Mountains provided an important refuge for *A. nanchuanensis* during Quaternary climate fluctuations. Genetic analysis revealed that the NHZ population not only had a special genetic background but also had specific differentiation in defense-related genes. The analysis also indicated that this species has an abundant genetic load, especially in the genes related to the early development and immune function of plants. Based on the study results, we divided the populations of A. nanchuanensis into four conservation units and proposed a number of practical management suggestions for each conservation unit of this critically endangered species.

## Data availability statement

The datasets presented in this study can be found in online repositories. The names of the repository/repositories and accession number(s) can be found below: NCBI, PRJNA948357 (SRA).

## Author contributions

HD designed the research. CX, BW and JZ collected the plant materials. CX, YZ and TX performed the research and analysed the data. CX and HZ wrote the paper. MK, XZ and HD revised the manuscript. All authors contributed to the article and approved the submitted version.

## References

[B1] Ackerly D. D. Loarie S. R. Cornwell W. K. Weiss S. B. Hamilton H. Branciforte R.. (2010). The geography of climate change: Implications for conservation biogeography. Divers. Distrib. 16, 476–487. doi: 10.1111/j.1472-4642.2010.00654.x

[B2] Alexander D. H. Novembre J. Lange K. (2009). Fast model-based estimation of ancestry in unrelated individuals. Genome Res. 19 (9), 1655–1664. doi: 10.1101/gr.094052.109 19648217 PMC2752134

[B3] Amtmann A. Troufflard S. Armengaud P. (2008). The effect of potassium nutrition on pest and disease resistance in plants. Physiol. Plant 133 (4), 682–691. doi: 10.1111/j.1399-3054.2008.01075.x 18331404

[B4] Axelrod D. I. Al-Shehbaz I. Raven P. H. (1998). “History of the modern flora of China,” in Floristic characteristics and diversity of East Asia plants. Eds. Zhang A. L. Wu S. G. (Beijing: China Higher Education Press), 43–55.

[B5] Barber Q. E. Nielsen S. E. Hamann A. (2016). Assessing the vulnerability of rare plants using climate change velocity, habitat connectivity, and dispersal ability: A case study in Alberta, Canada. Reg. Environ. Change 16, 1433–1441. doi: 10.1007/s10113-015-0870-6

[B6] Boria R. A. Olson L. E. Goodman S. M. Anderson R. P. (2014). Spatial filtering to reduce sampling bias can improve the performance of ecological niche models. Ecol. Modell. 275, 73–77. doi: 10.1016/j.ecolmodel.2013.12.012

[B7] Brown J. L. Hill D. J. Dolan A. M. Carnaval A. C. Haywood A. M. (2018). PaleoClim, high spatial resolution paleoclimate surfaces for global land areas. Sci. Data. 5, 180254. doi: 10.1038/sdata.2018.254 30422125 PMC6233254

[B8] Chen Y. X. Chen Y. S. Shi C. M. Huang Z. B. Zhang Y. Li S. K.. (2018). SOAPnuke: a MapReduce acceleration-supported software for integrated quality control and preprocessing of high-throughput sequencing data. Gigascience 7 (1), 1–6. doi: 10.1093/gigascience/gix120 PMC5788068 29220494

[B9] Chen C. J. Chen H. Zhang Y. Thomas H. R. Frank M. H. He Y. H.. (2020). TBtools: an integrative toolkit developed for interactive analyses of big biological data. Mol. Plant 13 (8), 1194–1202. doi: 10.1016/j.molp.2020.06.009 32585190

[B10] Cingolani P. Platts A. Wang L. Coon M. Nguyen T. Wang L.. (2012). A program for annotating and predicting the effects of single nucleotide polymorphisms, SnpEff: SNPs in the genome of *Drosophila melanogaster* strain w1118; iso-2; iso-3. Fly 6 (2), 80–92. doi: 10.4161/fly.19695 22728672 PMC3679285

[B11] Colleoni F. Wekerle C. Naslund J. O. Brandefelt J. Masina S. (2016). Constraint on the penultimate glacial maximum Northern Hemisphere ice topography (140 kyrs BP). Quat. Sci. Rev. 137, 97–112. doi: 10.1016/j.quascirev.2016.01.024

[B12] Corbett-Detig R. B. Hartl D. L. Sackton T. B. (2015). Natural selection constrains neutral diversity across a wide range of species. PloS Biol. 13 (4), e1002112. doi: 10.1371/journal.pbio.1002112 25859758 PMC4393120

[B13] Danecek P. Auton A. Abecasis G. Albers C. A. Banks E. DePristo M. A.. (2011). The variant call format and VCFtools. Bioinformatics 27 (15), 2156–2158. doi: 10.1093/bioinformatics/btr330 21653522 PMC3137218

[B14] Danecek P. Bonfield J. K. Liddle J. Marshall J. Ohan V. Pollard M. O.. (2021). Twelve years of SAMtools and BCFtools. GigaScience 10 (2), giab008. doi: 10.1093/gigascience/giab008 33590861 PMC7931819

[B15] Dawson T. P. Jackson S. T. House J. I. Prentice I. C. Mace G. M. (2011). Beyond predictions: biodiversity conservation in a changing climate. Science 332 (6025), 53–58. doi: 10.1126/science.1200303 21454781

[B16] Deng H. P. (2019). Biodiversity of Chongqing Jinfoshan National Nature Reserve (Beijing: Science Press).

[B17] Dickinson L. Noble H. Gardner E. Puad A. S. A. Zakaria W. N. F. W. Zerega N. J. C. (2020). Genetic diversity and structure of the critically endangered *Artocarpus annulatus*, a crop wild relative of jackfruit (*A. heterophyllus*). PeerJ 8, e9897. doi: 10.7717/peerj.9897 33005490 PMC7513743

[B18] Ding B. Hua B. Wen H. J. Deng H. P. Ding X. (2014). On floristic study and scientific name revision of seed type specimens distributed in Jinfo Mountains Nature Reserve. J. Southwest Norm. Univ. (Nat. Sci. Edit.) 39 (12), 47–52. doi: 10.13718/j.cnki.xsxb.2014.12.010

[B19] Doyle J. J. Doyle J. L. (1987). A rapid DNA isolation procedure for small quantities of fresh leaf tissue. Phytochem. Bull. 19 (1), 11–15. doi: 10.1016/0031-9422(80)85004-7

[B20] Finn R. D. Clements J. Arndt W. Miller B. L. Wheeler T. J. Schreiber F.. (2015). HMMER web server: 2015 update. Nucleic Acids Res. 43 (W1), W30–W38. doi: 10.1093/nar/gkv397 25943547 PMC4489315

[B21] Fos S. Laguna E. Jiménez J. (2014). Plant Micro-Reserves in the Valencian Region (E of Spain): are we achieving the expected results? Passive conservation of relevant vascular plant species. Flora Mediterr. 24, 153–162. doi: 10.7320/FlMedit24.153

[B22] Francis R. M. (2017). Pophelper: an R package and web app to analyse and visualize population structure. Mol. Ecol. Resour. 17, 27–32. doi: 10.1111/1755-0998.12509 26850166

[B23] Funk W. C. McKay J. K. Hohenlohe P. A. Allendorf F. W. (2012). Harnessing genomics for delineating conservation units. Trends Ecol. Evol. 27 (9), 489–496. doi: 10.1016/j.tree.2012.05.012 22727017 PMC4185076

[B24] Gao X. X. Liu J. Huang Z. H. (2022). The impact of climate change on the distribution of rare and endangered tree *Firmiana kwangsiensis* using the Maxent modeling. Ecol. Evol. 12 (8), e9165. doi: 10.1002/ece3.9165 35919389 PMC9336174

[B25] Gardner E. M. Johnson M. G. Pereira J. T. Puad A. S. A. Arifiani D. Sahromi. (2021). Paralogs and off-target sequences improve phylogenetic resolution in a densely sampled study of the breadfruit genus (*Artocarpus*, moraceae). Syst. Biol. 70 (3), 558–575. doi: 10.1093/sysbio/syaa073 PMC8048387 32970819

[B26] Gaut B. S. Morton B. R. McCaig B. C. Clegg M. T. (1996). Substitution rate comparisons between grasses and palms: synonymous rate differences at the nuclear gene *Adh* parallel rate differences at the plastid gene *rbcL* . Proc. Natl. Acad. Sci. U. S. A. 93 (19), 10274–10279. doi: 10.1073/pnas.93.19.10274 8816790 PMC38374

[B27] Gershenzon J. Dudareva N. (2007). The function of terpene natural products in the natural world. Nat. Chem. Biol. 3, 408–414. doi: 10.1038/nchembio.2007.5 17576428

[B28] Goodstein D. M. Shu S. Q. Howson R. Neupane R. Hayes R. D. Fazo J.. (2012). Phytozome: a comparative platform for green plant genomics. Nucleic. Acids Res. 40 (D1), D1178–D1186. doi: 10.1093/nar/gkr944 22110026 PMC3245001

[B29] Hazzouri K. M. Flowers J. M. Visser H. J. Khierallah H. S. M. Rosas U. Pham G. M.. (2015). Whole genome re-sequencing of date palms yields insights into diversification of a fruit tree crop. Nat. Commun. 6, 8824. doi: 10.1038/ncomms9824 26549859 PMC4667612

[B30] He L. (2014). Study on the morphological characteristics, modular biomass allocation and photosynthetic characteristics of Artocarpus nanchuanensis seedlings. (Chongqing: Southwest University).

[B31] He J. Y. Bao S. F. Deng J. H. Li Q. F. Ma S. Y. Liu Y. R.. (2022a). A chromosome-level genome assembly of *Artocarpus nanchuanensis* (Moraceae), an extremely endangered fruit tree. GigaScience 11, 1–13. doi: 10.1093/gigascience/giac042 PMC9197682 35701376

[B32] He J. Y. Bao S. F. Deng J. H. Li Q. F. Ma S. Y. Liu Y. R.. (2022b). Supporting data for “A chromosome-level genome assembly of *Artocarpus nanchuanensis* (Moraceae), an extremely endangered fruit tree”. GigaSci. Database. doi: 10.5524/102200 PMC9197682 35701376

[B33] Hofreiter M. Stewart J. (2009). Ecological change, range fluctuations and population dynamics during the Pleistocene. Curr. Biol. 19, 584–594. doi: 10.1016/j.cub.2009.06.030 19640497

[B34] Hohenlohe P. A. Funk W. C. Rajora O. P. (2021). Population genomics for wildlife conservation and management. Mol. Ecol. 30 (1), 62–82. doi: 10.1111/mec.15720 33145846 PMC7894518

[B35] Hu J. Y. Hao Z. Q. Frantz L. Wu S. F. Chen W. Jiang Y. F.. (2020). Genomic consequences of population decline in critically endangered pangolins and their demographic histories. Natl. Sci. Rev. 7 (4), 798–814. doi: 10.1093/nsr/nwaa031 34692098 PMC8288997

[B36] Hu J. Y. Kuang W. M. Wu H. Yu L. (2021). Genomic genetic load analysis. Bio101, e1010600. doi: 10.21769/BioProtoc.1010600

[B37] Huang H. W. (2011). Plant diversity and conservation in China: Planning a strategic bioresource for a sustainable future. Bot. J. Linn. Soc 166, 282–300. doi: 10.1111/j.1095-8339.2011.01157.x 22059249

[B38] Huang J. Liang X. M. Xuan Y. K. Geng C. Y. Li Y. X. Lu H. R.. (2018). BGISEQ-500 WGS library construction. (protocols.io). doi: 10.17504/protocols.io.ps5dng6

[B39] Huang H. W. Zhang Z. (2012). Current status and prospects of ex situ cultivation and conservation of plants in China. Biodivers. Sci. 20 (5), 559–571. doi: 10.3724/SP.J.1003.2012.13124

[B40] Jangjoo M. Matter S. F. Roland J. Keyghobadi N. (2016). Connectivity rescues genetic diversity after a demographic bottleneck in a butterfly population network. Proc. Natl. Acad. Sci. U. S. A. 113, 10914–10919. doi: 10.1073/pnas.1600865113 27621433 PMC5047165

[B41] Jia Q. Brown R. Köllner T. G. Fu J. Chen X. Wong G. K.. (2022). Origin and early evolution of the plant terpene synthase family. Proc. Natl. Acad. Sci. U. S. A. 119 (15), e2100361119. doi: 10.1073/pnas.2100361119 35394876 PMC9169658

[B42] Kessler A. (2001). Defensive function of herbivore-induced plant volatile emissions in nature. Sci. 291 (5511), 2141–2144. doi: 10.1126/science.291.5511.2141 11251117

[B43] Laguna E. Fos S. Jimenez J. Volis S. (2016). Role of micro-reserves in conservation of endemic, rare and endangered plants of the Valencian region (Eastern Spain). Isr. J. Plant Sci. 63 (4), 320–332. doi: 10.1080/07929978.2016.1256131

[B44] Li H. (2013). Aligning sequence reads, clone sequences and assembly contigs with BWA-MEM. arXiv 1303, 3997. doi: 10.48550/arXiv.1303.3997

[B45] Li H. Durbin R. (2011). Inference of human population history from individual whole-genome sequences. Nature 475 (7357), 493–496. doi: 10.1038/nature10231 21753753 PMC3154645

[B46] Li J. L. Milne R. I. Ru D. F. Miao J. B. Tao W. J. Zhang L.. (2020). Allopatric divergence and hybridization within *Cupressus chengiana* (Cupressaceae), a threatened conifer in the northern Hengduan Mountains of western China. Mol. Ecol. 29 (7), 1250–1266. doi: 10.1111/mec.15407 32150782

[B47] Lin X. G. Feng C. Lin T. Harris A. J. Li Y. Z. Kang M. (2022). Jackfruit genome and population genomics provide insights into fruit evolution and domestication history in China. Hortic. Res. 9, uhac173. doi: 10.1093/hr/uhac173 36204202 PMC9533223

[B48] Lin W. W. Li B. Lu D. P. Chen S. X. Zhu N. He P.. (2014). Tyrosine phosphorylation of protein kinase complex BAK1/BIK1 mediates Arabidopsis innate immunity. Proc. Natl. Acad. .Sci. U. S. A. 111, 3632–3637. doi: 10.1073/pnas.1318817111 24532660 PMC3948311

[B49] Liu J. Liu B. Chen S. F. Gong B. Q. Chen L. J. Zhou Q.. (2018). A tyrosine phosphorylation cycle regulates fungal activation of a plant receptor ser/thr kinase. Cell Host Microbe 23, 241–253. doi: 10.1016/j.chom.2017.12.005 29396039

[B50] López-Pujol J. Zhang F. M. Sun H. Q. Ying T. S. Ge S. (2011). Centres of plant endemism in China: Places for survival or for speciation? J. Biogeogr. 38, 1267–1280. doi: 10.1111/j.1365-2699.2011.02504.x

[B51] Ma H. Liu Y. B. Liu D. T. Sun W. B. Liu X. F. Wan Y. M.. (2021). Chromosome-level genome assembly and population genetic analysis of a critically endangered rhododendron provide insights into its conservation. Plant J. 107, 1533–1545. doi: 10.1111/tpj.15399 34189793

[B52] Ma Y. P. Liu D. T. Wariss H. M. Zhang R. G. Tao L. D. Milne R. I.. (2022). Demographic history and identification of threats revealed by population genomic analysis provide insights into conservation for an endangered maple. Mol. Ecol. 31, 767–779. doi: 10.1111/mec.16289 34826164

[B53] Macho A. P. Lozano-Durán R. Zipfel C. (2015). Importance of tyrosine phosphorylation in receptor kinase complexes. Trends Plant Sci. 20 (5), 269–272. doi: 10.1016/j.tplants.2015.02.005 25795237

[B54] Macho A. P. Schwessinger B. Ntoukakis V. Brutus A. Segonzac C. Roy S.. (2014). A bacterial tyrosine phosphatase inhibits plant pattern recognition receptor activation. Science 343, 1509–1512. doi: 10.1126/science.1248849 24625928

[B55] Mattila A. L. Duplouy A. Kirjokangas M. Lehtonen R. Rastas P. Hanski I. (2012). High genetic load in an old isolated butterfly population. Proc. Natl. Acad. .Sci. U. S. A. 109, E2496–E2505. doi: 10.1073/pnas.1205789109 22908265 PMC3443129

[B56] Nei M. Li W. H. (1979). Mathematical model for studying genetic variation in terms of restriction endonucleases. Proc. Natl. Acad. .Sci. U. S. A. 76 (10), 5269–5273. doi: 10.1073/pnas.76.10.5269 291943 PMC413122

[B57] Nguyen L. T. Schmidt H. A. von Haeseler A. Minh B. Q. (2015). IQ-TREE: A fast and effective stochastic algorithm for estimating maximum-likelihood phylogenies. Mol. Biol. Evol. 32 (1), 268–274. doi: 10.1093/molbev/msu300 25371430 PMC4271533

[B58] Patil A. B. Vajja S. S. Raghavendra S. Satish B. N. Kushalappa C. G. Vijay N. (2022). Jack of all trades: Genome assembly of Wild Jack and comparative genomics of *Artocarpus* . Front. Plant Sci. 13. doi: 10.3389/fpls.2022.1029540 PMC9791056 36578332

[B59] Phillips S. J. Anderson R. P. Schapire R. E. (2006). Maximum entropy modelling of species’ geographic distributions. Ecol. Model. 12, 231–259. doi: 10.1016/j.ecolmodel.2005.03.026

[B60] Pimm S. L. (2008). Biodiversity: climate change or habitat loss—which will kill more species? Curr. Biol. 18 (3), R117–R119. doi: 10.1016/j.cub.2007.11.055 18269905

[B61] Purcell S. Neale B. Todd-Brown K. Thomas L. Ferreira M. A. R. Bender D.. (2007). Plink: A tool set for whole-genome association and population-based linkage analyses. Am. J. Hum. Genet. 81 (3), 559–575. doi: 10.1086/519795 17701901 PMC1950838

[B62] Qian S. H. Yang Y. Tang C. Q. Momohara A. Yi S. R. Ohsawa M. (2016). Effective conservation measures are needed for wild *Cathaya argyrophylla* populations in China: Insights from the population structure and regeneration characteristics. For. Ecol. Manage. 361, 358–367. doi: 10.1016/j.foreco.2015.11.041

[B63] Qin A. L. Liu B. Guo Q. S. Bussmann R. W. Ma F. Q. Jian Z. L.. (2017a). Maxent modeling for predicting impacts of climate change on the potential distribution of *Thuja sutchuenensis* Franch., an extremely endangered conifer from southwestern China. Glob. Ecol. Conserv. 10, 139–146. doi: 10.1016/j.gecco.2017.02.004

[B64] Qin H. N. Yang Y. Dong S. Y. He Q. Jia Y. Zhao L. N.. (2017b). Threatened species list of China’s higher plants. Biodivers. Sci. 25 (7), 696–744. doi: 10.17520/biods.2017144

[B65] Qiu Y. X. Fu C. X. Comes H. P. (2011). Plant molecular phylogeography in China and adjacent regions: tracing the genetic imprints of Quaternary climate and environmental change in the world’s most diverse temperate flora. Mol. Phylogenet. Evol. 59, 225–244. doi: 10.1016/j.ympev.2011.01.012 21292014

[B66] Richter A. Seidl-Adams I. Köllner T. G. Schaff C. Tumlinson J. H. Degenhardt J. (2015). A small, diferentially regulated family of farnesyl diphosphate synthases in maize (*Zea mays*) provides farnesyl diphosphate for the biosynthesis of herbivore-induced sesquiterpenes. Planta 241, 1351–1361. doi: 10.1007/s00425-015-2254-z 25680349

[B67] Shi X. T. Long Y. He F. Zhang C. Y. Wang R. Y. Zhang T.. (2018). The fungal pathogen *Magnaporthe oryzae* suppresses innate immunity by modulating a host potassium channel. PloS Pathog. 14 (1), e1006878. doi: 10.1371/journal.ppat.1006878 29385213 PMC5809103

[B68] Swets J. A. (1988). Measuring the accuracy of diagnostic systems. Science 240 (4857), 1285–1293. doi: 10.1126/science.3287615 3287615

[B69] Tajima F. (1983). Evolutionary relationship of DNA sequences in finite populations. Genetics 105, 437–460. doi: 10.1093/genetics/105.2.437 6628982 PMC1202167

[B70] Tan X. M. Zhou Y. Q. Deng A. G. Liu Y. T. Peng Y. Zhou X. Z. (2015). Study on seedling techniques of *Artocarpus nanchuanensis* . Agr. Sci. Tech. 16 (12), 2754–2757. doi: 10.16175/j.cnki.1009-4229.2015.12.037

[B71] Tang C. Q. Matsui T. Ohashi H. Dong Y. F. Momohara A. Herrando-Moraira S.. (2018). Identifying long-term stable refugia for relict plant species in East Asia. Nat. Commun. 9, 4488. doi: 10.1038/s41467-018-06837-3 30367062 PMC6203703

[B72] Tang C. Q. Yang Y. Ohsawa M. Yi S. R. Momohara A. Su W. H.. (2012). Evidence for the persistence of wild *Ginkgo biloba* (Ginkgoaceae) populations in the Dalou Mountains, southwestern China. Am. J. Bot. 99 (8), 1408–1414. doi: 10.3732/ajb.1200168 22847538

[B73] Thomas C. D. Cameron A. Green R. E. Bakkenes M. Beaumont L. J. Collingham Y. C.. (2004). Extinction risk from climate change. Nature 427, 145–148. doi: 10.1038/nature02121 14712274

[B74] Wang H. W. Ge S. (2006). Phylogeography of the endangered *Cathaya argyrophylla* (Pinaceae) inferred from sequence variation of mitochondrial and nuclear DNA. Mol. Ecol. 15, 4109–4122. doi: 10.1111/j.1365-294X.2006.03086.x 17054506

[B75] Wang J. Street N. R. Scofield D. G. Ingvarsson P. K. (2016). Variation in linked selection and recombination drive genomic divergence during allopatric speciation of European and American aspens. Mol. Biol. Evol. 33 (7), 1754–1767. doi: 10.1093/molbev/msw051 26983554 PMC4915356

[B76] Wang Y. Wu W. H. (2015). Genetic approaches for improvement of the crop potassium acquisition and utilization efficiency. Curr. Opin. Plant Biol. 25, 46–52. doi: 10.1016/j.pbi.2015.04.007 25941764

[B77] Westerhold T. Marwan N. Drury A. J. Liebrand D. Agnini C. Anagnostou E.. (2020). An astronomically dated record of Earth’s climate and its predictability over the last 66 million years. Science 369 (6509), 1383–1388. doi: 10.1126/science.aba685 32913105

[B78] Wickham H. (2016). ggplot2—elegant graphics for data analysis. 2nd edn (New York: Springer).

[B79] Wu X. Q. Xu G. B. Liang Y. Shen X. B. (2013). Genetic diversity of natural and planted populations of *Tsoongiodendron odorum* from the Nanling Mountains. Biodivers. Sci. 21 (1), 71–79. doi: 10.3724/SP.J.1003.2013.09138

[B80] Wu Z. Y. Zhang X. S. (1989). Some new taxa of Moraceae in China. Acta Botanica Yunnanica 11, 24–34.

[B81] Xu B. Liao M. Deng H. N. Yan C. C. Lv Y. Y. Gao Y. D.. (2022). Chromosome-level *de novo* genome assembly and whole-genome resequencing of the threatened species *Acanthochlamys bracteata* (Velloziaceae) provide insights into alpine plant divergence in a biodiversity hotspot. Mol. Ecol. Resour. 22, 1582–1595. doi: 10.1111/1755-0998.13562 34837470

[B82] Xu Y. Zang R. G. (2022). Theoretical and practical research on conservation of Wild Plants with Extremely Small Populations in China. Biodivers. Sci. 30, 22505. doi: 10.17520/biods.2022505

[B83] Xue T. T. Yang X. D. Liu Q. Qin F. Zhang W. D. Janssens S. B.. (2022). Integration of hotspot identification, gap analysis, and niche modeling supports the conservation of Chinese threatened higher plants. J. Syst. Evol. 1–16. doi: 10.1111/jse.12901

[B84] Yi H. Q. Wang J. Y. Wang J. Rausher M. Kang M. (2022). Genomic insights into inter- and intraspecific mating system shifts in *Primulina* . Mol. Ecol. 31 (22), 5699–5713. doi: 10.1111/mec.16706 36178058

[B85] Yu Z. M. Zhao C. H. Zhang G. H. Teixeira da Silva J. A. Duan J. (2020). Genome-Wide identification and expression profile of TPS gene family in *dendrobium officinale* and the role of *doTPS10* in linalool biosynthesis. Int. J. Mol. Sci. 21, 5419. doi: 10.3390/ijms21155419 32751445 PMC7432446

[B86] Zhang F. C. (2017). Nature Small-reserves helps biodiversity conservation. China Environ. 000 (010), 30–33.

[B87] Zhao Y. P. Fan G. Yin P. P. Sun S. Li N. Hong X. N.. (2019). Resequencing 545 ginkgo genomes across the world reveals the evolutionary history of the living fossil. Nat. Commun. 10, 4201. doi: 10.1038/s41467-019-12133-5 31519986 PMC6744486

[B88] Zhao L. Zhao X. J. Francis F. Liu Y. (2021). Genome−wide identifcation and characterization of the TPS gene family in wheat (*Triticum aestivum* L.) and expression analysis in response to aphid damage. Acta Physiol. Plant 43, 64. doi: 10.1007/s11738-021-03236-y

[B89] Zu K. L. Wang Z. H. Lenoir J. Shen Z. H. Chen F. S. Shrestha N. (2022). Different range shifts and determinations of elevational redistributions of native and non-native plant species in Jinfo Mountain of subtropical China. Ecol. Indic. 145, 109678. doi: 10.1016/j.ecolind.2022.109678

[B90] Zu K. L. Zhang C. C. Chen F. S. Zhang Z. Y. Ahmad S. Nabi G. (2023). Latitudinal gradients of angiosperm plant diversity and phylogenetic structure in China’s nature reserves. Glob. Ecol. Conserv. 42, e02403. doi: 10.1016/j.gecco.2023.e02403

