# Supplementary material for: The genetic structure and demographic history revealed by whole-genome resequencing provide insights into conservation of critically endangered Artocarpus nanchuanensis
Source: Front Plant Sci. 2023 Jul 27;14:1224308. doi: 10.3389/fpls.2023.1224308 (PMC10415164; doi:10.3389/fpls.2023.1224308)
Supplement: Supplementary file 1 [file DataSheet_1.doc]

***Supplementary Material***

**The genetic structure and demographic history revealed by whole-genome resequencing provide insights into conservation of critically endangered *Artocarpus nanchuanensis* (Moraceae)**

**Changying Xia^1^, Youwei Zuo^1^, Tiantian Xue^2^, Ming Kang^3^, Huan Zhang^1^, Xiaoxia Zhang^2^, Binru Wang^1^, Jiabin Zhang^1^, Hongping Deng^1, 4*^**

*** Correspondence:** Hongping Deng: [denghp@swu.edu.cn](mailto:denghp@swu.edu.cn)

**Contents**

[Supplementary Figure 1. 3](#__RefHeading___Toc135078066)

[Supplementary Figure 2. 4](#__RefHeading___Toc135078067)

[Supplementary Figure 3 4](#__RefHeading___Toc135078068)

[Supplementary Figure 4. 5](#__RefHeading___Toc135078069)

[Supplementary Figure 5 6](#__RefHeading___Toc135078070)

[Supplementary Figure 6 6](#__RefHeading___Toc135078071)

[Supplementary Figure 7. 7](#__RefHeading___Toc135078072)

[Supplementary Figure 8 7](#__RefHeading___Toc135078073)

[Supplementary Figure 9 8](#__RefHeading___Toc135078074)


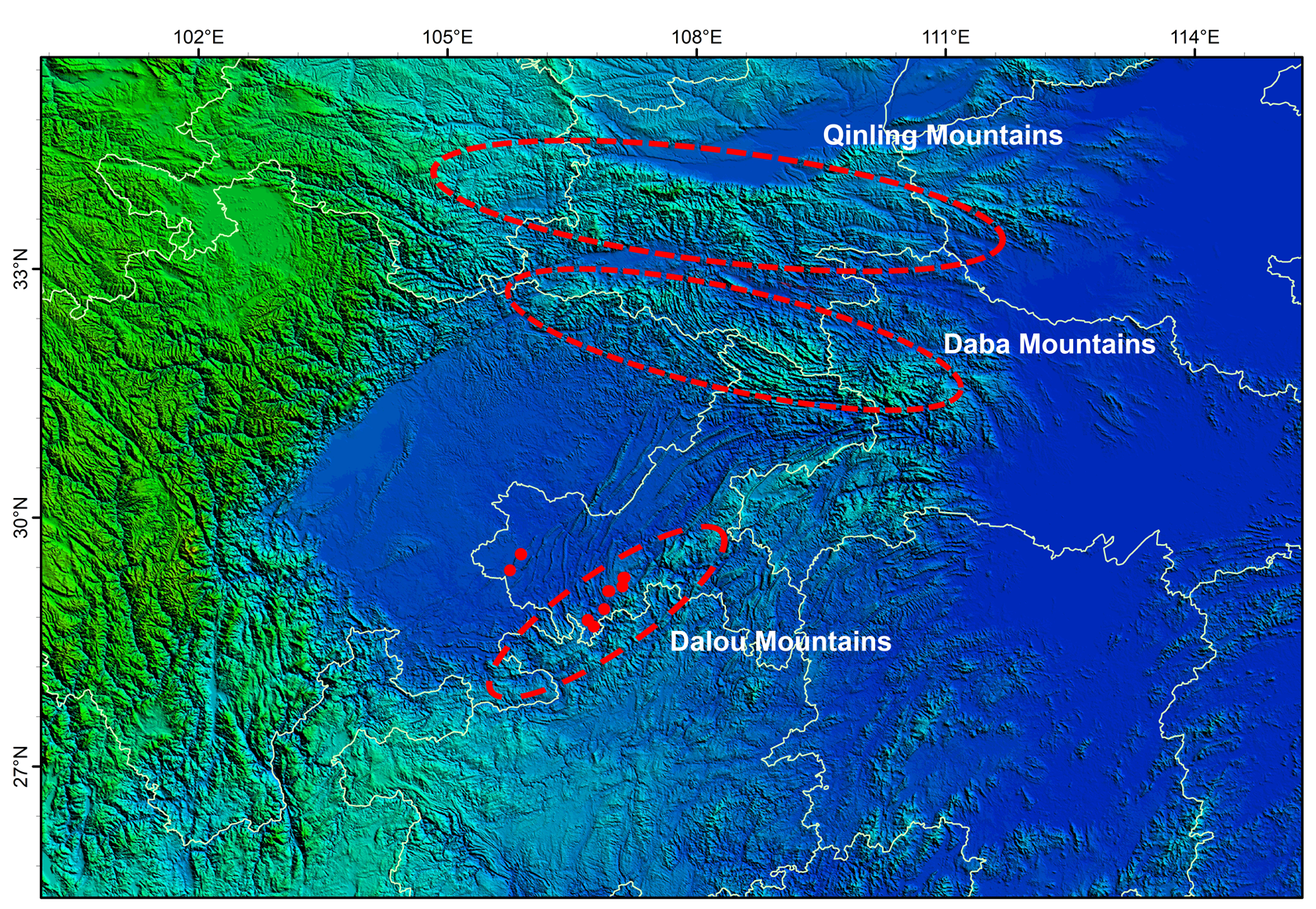


**Supplementary Figure 1.** The geographical distribution of the sampled populations of *Artocarpus nanchuanensis* and the main adjacent mountains.


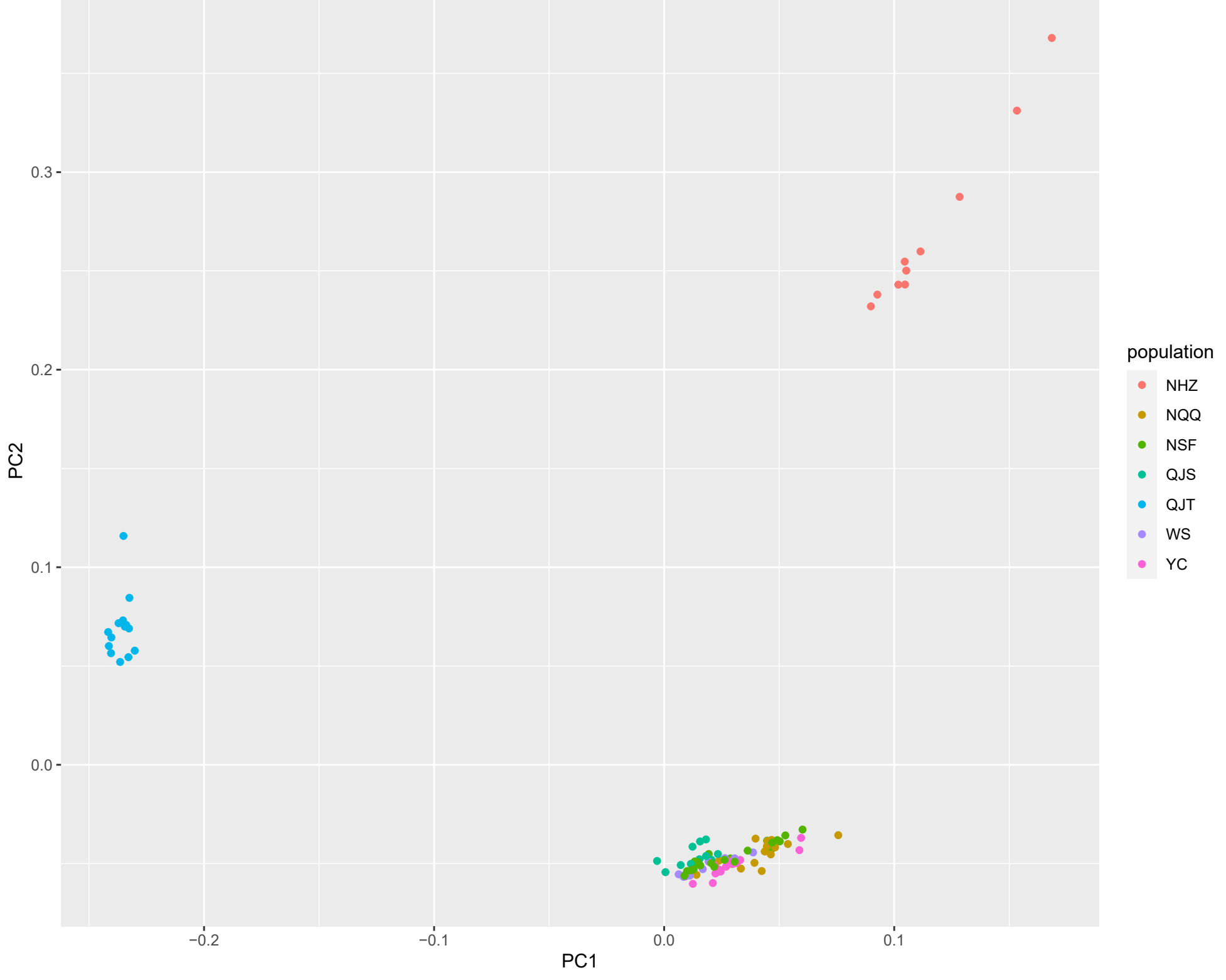


**Supplementary Figure 2.** Principal component analysis (PCA) plot for the 101 *A. nanchuanensis* individuals based on PC1and PC2.


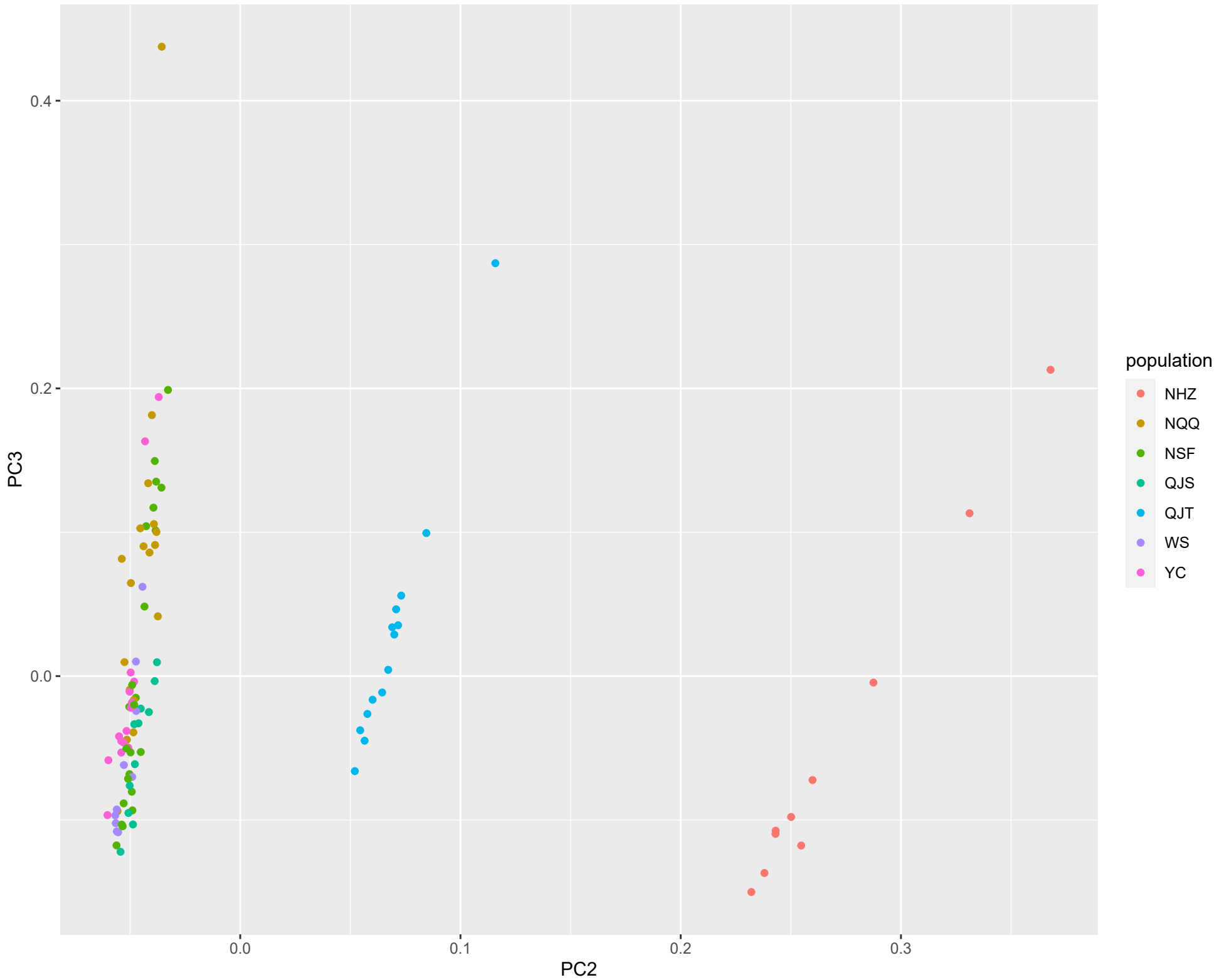


**Supplementary Figure 3.** Principal component analysis (PCA) plot for the 101 *A. nanchuanensis* individuals based on PC2 and PC3.

**
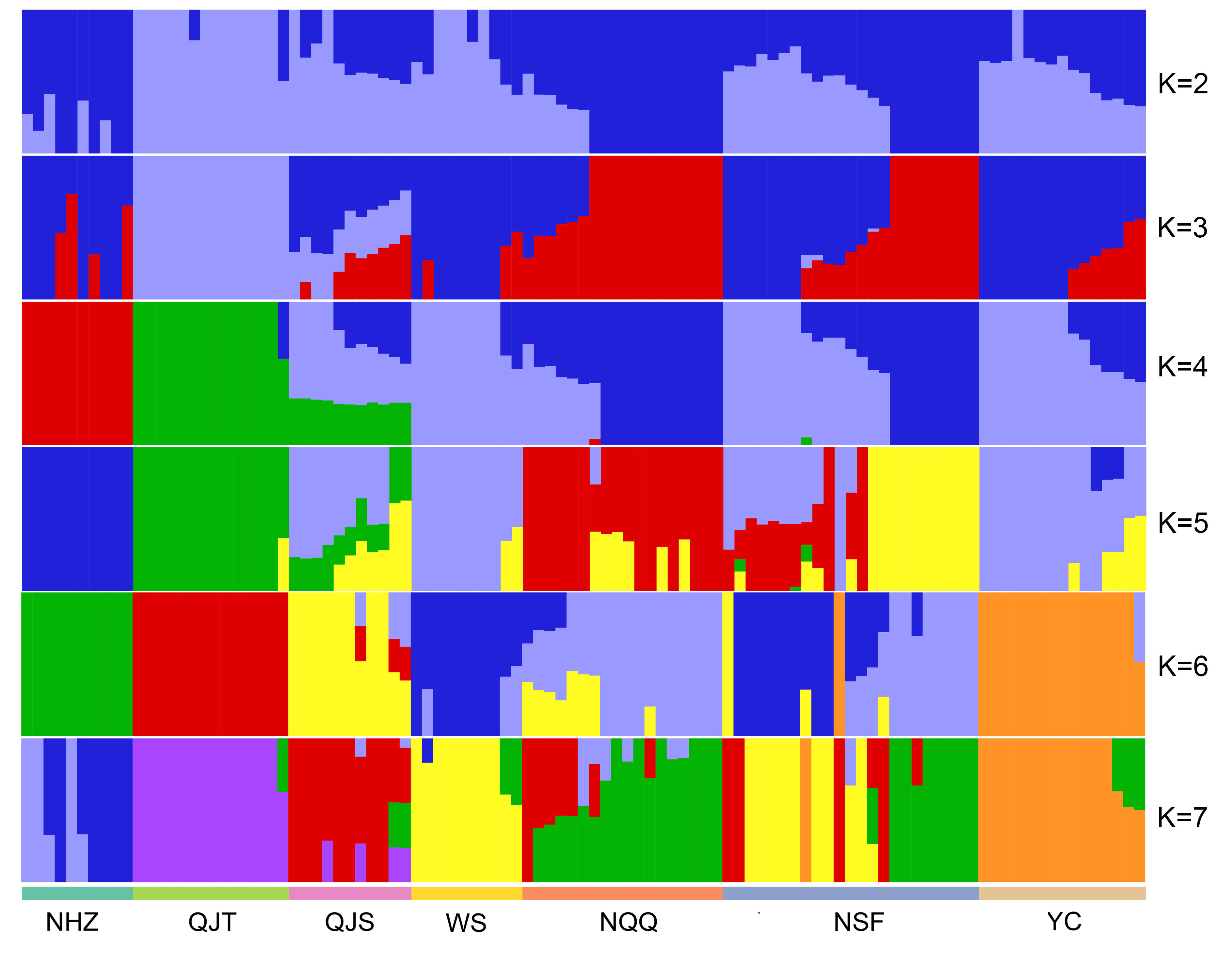
**

**Supplementary Figure 4.** Population structure of 101 *A. nanchuanensis* individuals based on K=2-7.


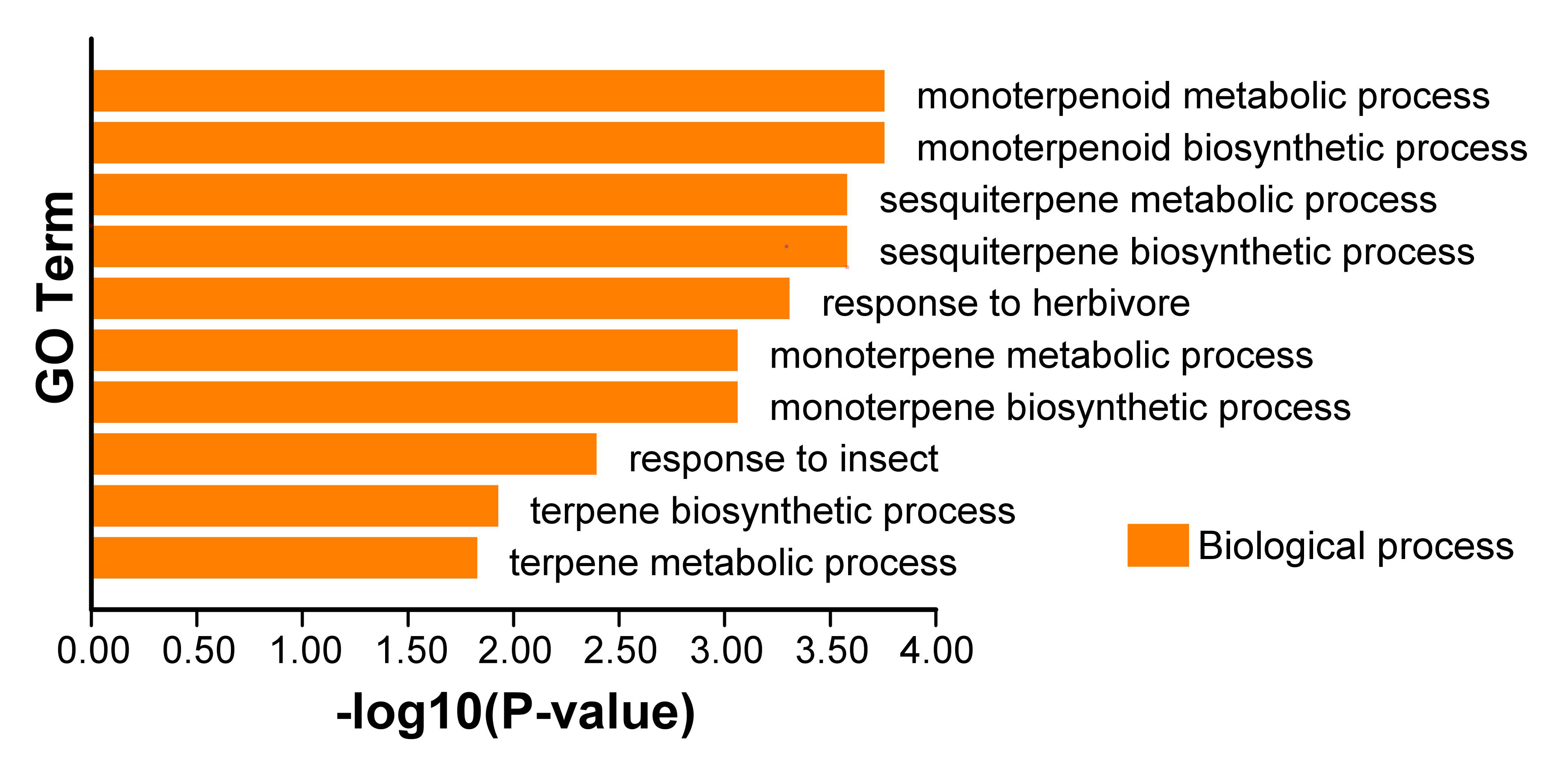


**Supplementary Figure 5.** The GO terms enrichment of the selected genes from NHZ population.


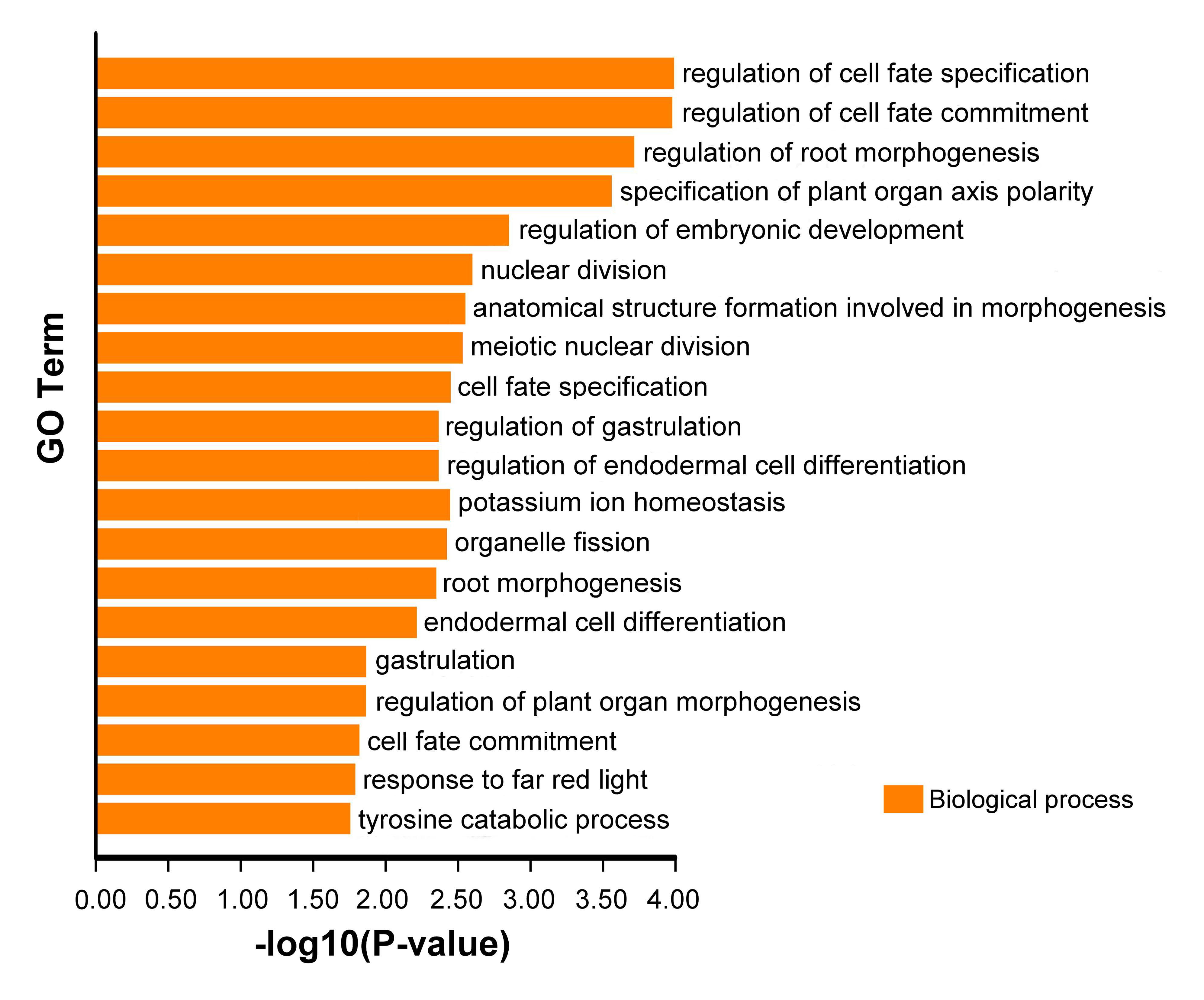


**Supplementary Figure 6.** The GO terms enrichment of genes containing homozygous loss of function mutations (LOF).


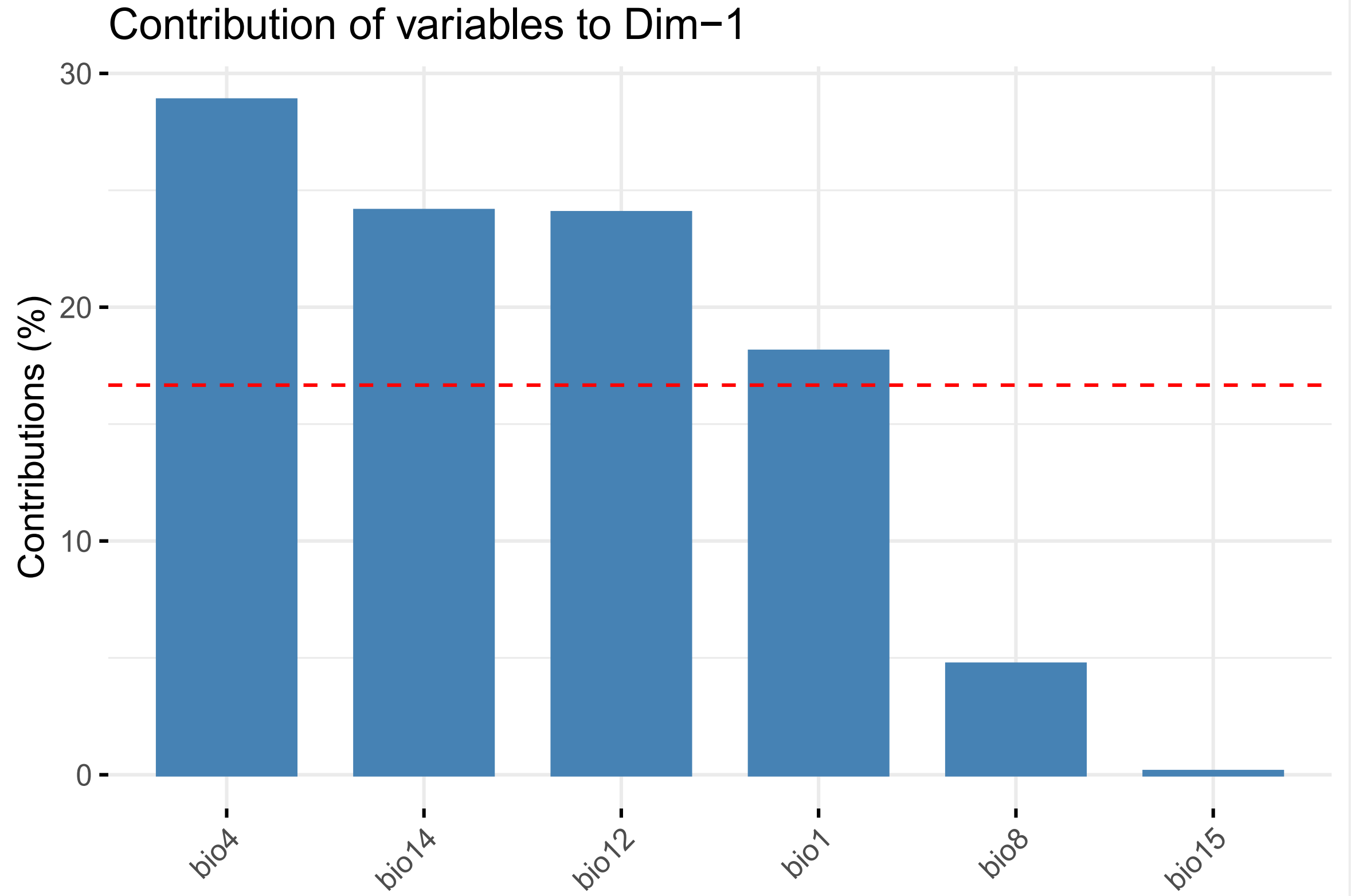


**Supplementary Figure 7.** Scree plot of the percentage of explained variance to dimension 1.


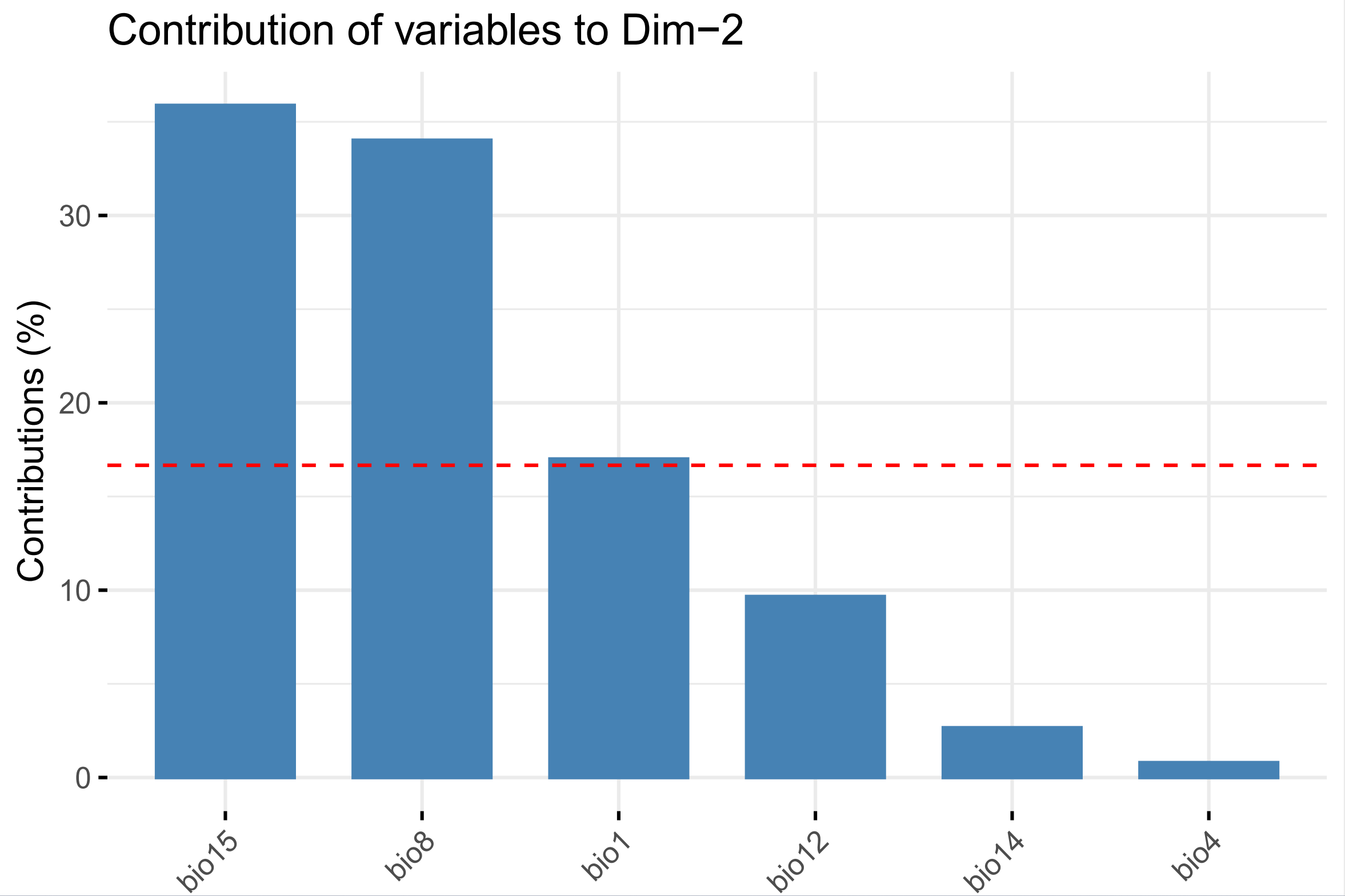


**Supplementary Figure 8.** Scree plot of the percentage of explained variance to dimension 2.


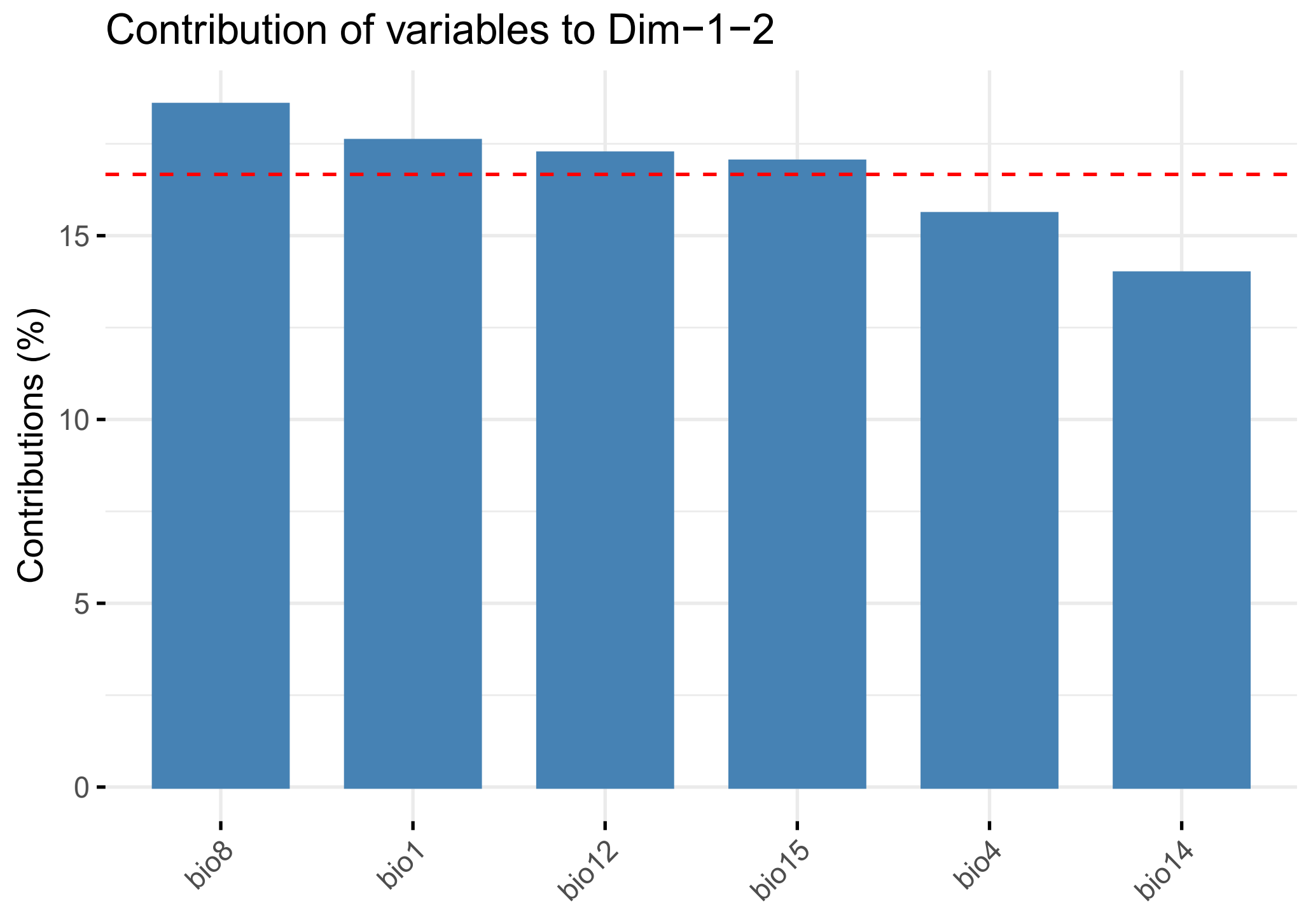


**Supplementary Figure 9.** Scree plot of the percentage of explained variance to dimensions 1 and 2.
